# From identification to forecasting: the potential of image recognition and artificial intelligence for aphid pest monitoring

**DOI:** 10.3389/fpls.2023.1150748

**Published:** 2023-07-19

**Authors:** Philipp Batz, Torsten Will, Sebastian Thiel, Tim Mark Ziesche, Christoph Joachim

**Affiliations:** ^1^ ALM – Adaptiv Lernende Maschinen – Gesellschaft mit beschränkter Haftung (GmbH), Nisterau, Germany; ^2^ Institute for Resistance Research and Stress Tolerance, Julius Kühn-Institute, Federal Research Centre for Cultivated Plants, Quedlinburg, Germany; ^3^ Institute for Plant Protection in Field Crops and Grassland, Julius Kühn-Institute, Federal Research Centre for Cultivated Plants, Braunschweig, Germany

**Keywords:** deep learning, convolutional neural network, integrated pest management, decision support systems, image based identification, applied entomology, transformer models

## Abstract

Insect monitoring has gained global public attention in recent years in the context of insect decline and biodiversity loss. Monitoring methods that can collect samples over a long period of time and independently of human influences are of particular importance. While these passive collection methods, e.g. suction traps, provide standardized and comparable data sets, the time required to analyze the large number of samples and trapped specimens is high. Another challenge is the necessary high level of taxonomic expertise required for accurate specimen processing. These factors create a bottleneck in specimen processing. In this context, machine learning, image recognition and artificial intelligence have emerged as promising tools to address the shortcomings of manual identification and quantification in the analysis of such trap catches. Aphids are important agricultural pests that pose a significant risk to several important crops and cause high economic losses through feeding damage and transmission of plant viruses. It has been shown that long-term monitoring of migrating aphids using suction traps can be used to make, adjust and improve predictions of their abundance so that the risk of plant viruses spreading through aphids can be more accurately predicted. With the increasing demand for alternatives to conventional pesticide use in crop protection, the need for predictive models is growing, e.g. as a basis for resistance development and as a measure for resistance management. In this context, advancing climate change has a strong influence on the total abundance of migrating aphids as well as on the peak occurrences of aphids within a year. Using aphids as a model organism, we demonstrate the possibilities of systematic monitoring of insect pests and the potential of future technical developments in the subsequent automated identification of individuals through to the use of case data for intelligent forecasting models. Using aphids as an example, we show the potential for systematic monitoring of insect pests through technical developments in the automated identification of individuals from static images (i.e. advances in image recognition software). We discuss the potential applications with regard to the automatic processing of insect case data and the development of intelligent prediction models.

## 1 Introduction

Aphids (Hemiptera: Aphidoidea) are among the most important insect pests of arable and horticultural crops. These soft-bodied, phytophagous insects mostly feed on phloem sap of plants using a piercing-sucking stylet (Dixon, 2012). There are more than 5,500 aphid species worldwide with approximately 250 species considered as economically relevant pest species (Blackman and Eastop, 2006; Favret, 2018). On a local scale, only a limited number of species are monitored regularly, whereas the composition and number of species collected within a year varies significantly between successive years with a trend of an increase in species numbers, as demonstrated for Great Britain and France between 1978 and 2000 (Hullé et al., 2010).

Migrating aphids are of special interest for agriculture in spring and fall, when they form initial infestation sites in fields, which can lead to crop yield losses by direct feeding damage due to nutrient withdrawal or through secretion of saliva as well accompanied by the transmission of phytopathogenic viruses (Robert et al., 2000; Girousse et al., 2005; Giordanengo et al., 2010). Direct feeding damage has been shown for e.g. the Bird cherry-oat aphid, *Rhopalosiphum padi*, in spring wheat with yield reduction of up to 20% (Voss et al., 1997) and Black bean aphids *Aphis fabae* in sugar beets can cause up to 50% reduction in root dry weight (Hurej and van der Werf, 1993). Average percentage losses have been estimated between 4% and 46% for potatoes and field beans, respectively (Tatchell, 1989). However, estimations of economic losses by direct feeding damage as a consequence of aphid infestation in the field are scarce.

Furthermore, aphids transmit viruses that can cause serious plant diseases (Stevens and Lacomme, 2017). Many phytopathogenic viruses use aphids as vectors, such as *Barley yellow dwarf virus* (BYDV) in e.g. barley and wheat, *Turnip yellows virus* in e.g. winter oilseed rape, *Potato virus Y* in potato and *Beet yellows virus* in sugar beet. In total, approximately 275 different viruses are vectored by 192 different aphid species (Nault, 1997) not including the recently described group of nanoviruses, which are transmitted by the pea aphid *Acyrthosiphon pisum* and infect legumes (Gaafar and Ziebell, 2020; Lal et al., 2020). Although many viruses have the potential to cause significant crop losses, a high percentage of economic losses are most likely to be attributed to individual fields or limited spatial areas. Regarding BYDV, a more recent estimation by an expert assessment estimates the economic losses of a BYDV infection for NW-Europe to be 3.26% per year (Savary et al., 2019).

In 2013, the European Union (EU) decided on a ban of three insecticides from the group of neonicotinoids for seed coating (clothianidin, imidacloprid and thiamethoxam) which hitherto was one of the most efficient and economic options to protect seedlings and young plants against early infestation by phytophagous insect pests, such as aphids, and insect-transmitted viruses (Verheggen et al., 2022). While exemptions for seed and soil treatment in certain cereals were possible between 2013 and 2018, since then the use of the mentioned neonicotinods is restricted to greenhouse uses only (European Union, 2018). In addition, the EU decided not to renew the authorization of thiacloprid, a commonly used insecticide of the same group, from the beginning of 2021 (Commission Implementing Regulation (EU) 2020/23, 2020). Due to the lack of equivalent alternatives, the control of insect pests and pest associated pathogens became more difficult. As a consequence, the pressure on agricultural yield stability is increasing as resistance to insecticides, e.g. pirimicarb belonging to the group of carbamates and pyrethroids, has been observed for several years in Germany, other European countries (Nauen and Elbert, 2003) as well as on a worldwide scale (Edwards et al., 2008). Studies on the Green peach aphid, *Myzus persicae*, in France indicate that the expansion of monocultures and the intensification of insecticide use lead to the selection for resistance against organophosphates and pyrethroids in the field (Zamoum et al., 2005). Selection of individual biotypes has also been observed in the English grain aphid *Sitobion avenae* in Great Britain (Llewellyn et al., 2003). Occasional prophylactic, large-scale or non-targeted insecticide applications are drivers of emerging resistance in a number of different aphid species (Bhatia et al., 2011), but also with the number of approved insecticidal active substances decreasing the risk of resistance development against the remaining substances increases (Després et al., 2007).

It becomes evident that this development, i.e. the reduction of available active ingredients in plant protection products in combination with increasing insect resistance against remaining insecticides, increases the potential of aphid induced yield losses in agricultural production systems. To counteract these losses, continuous monitoring of aphids in agricultural landscapes will become essential for yield stability, since aphid abundances and diversity as well as viral load of aphids are factors that may affect crop growth and plant virus transmission. A high temporal resolution of the crops’ pest pressure is of great economic importance to farmers. Additionally, monitoring data will improve the understanding of the impact of climate change on pest development and migration, hence building a basis to improve forecasting models. This kind of monitoring could be achieved by comprehensive mass trapping using yellow pan traps at local and suction traps at regional scales (e.g. Harrington et al., 2007; Kirchner et al., 2013). The biggest constraints for the utilization of such traps are, however, the limited availability of expert knowledge for species identification and the time-consuming manual identification process.

Recent advantages in artificial intelligence (AI) enabled the development of automated solutions for insect identification and classification. By efficiently processing large amounts of images without human intervention, these methods offer a possible solution in the context of time sensitive analysis and the shortage of adequate expertise mentioned above. In this review, we use aphids as a model organism to evaluate the potential of image recognition and AI in agricultural pest monitoring. We give an overview of the current methods of insect monitoring, possibilities of species identification and demonstrate how image recognition could support the identification process of high sample quantities from mass trap catches. We conclude by showing how data generated this way could be utilized for forecasting models.

## 2 Aphid monitoring

### 2.1 Aphid sampling

For targeted protection of agricultural field crops, it is mandatory to assess the temporal and spatial occurrence of harmful organisms (Barzman et al., 2015), i.e. insect monitoring is fundamental for the development of control strategies following the guidelines of integrated pest management. This way, prophylactic applications of plant protection products can be avoided, since beside ecological aspects, prophylactic applications do not guarantee a reduction in pest pressure and can be economically questionable, as aphid occurrence can vary greatly from year to year, with seasonal and regional variations in composition and abundance (e.g. Dixon, 2012; Luquet et al., 2019; Bell et al., 2020). Recent data also indicate that regional synchrony between aphid populations will decrease as a consequence of climate change (Sheppard et al., 2016), making small scale monitoring even more important for addressing regional fluctuations in pest occurrence.

For agricultural monitoring purposes, a wide range of active or passive sampling techniques enables aphid monitoring either on the crop or *via* aerial sampling (Harrington and Hullé, 2017). In order to assess the potential benefits of AI on samples from these methods, knowledge of the common methods used to monitor aphids is essential and is provided below:

Crop sampling is best suited to determine aphid infestation rates and aphid abundance at a field scale. Although this sampling method is outside the focus of this review, we give a brief summary for the sake of completeness. In everyday agricultural practice, visual aphid observation or *in-situ* aphid counts on plants or plant parts are carried out by an on-site human observer and allow farmers, advisors and growers to directly assess local, economic relevant thresholds and apply control strategies according to them. These strategies can, however, only be adequately applied on fields where the aphid pest pressure has been assessed, since crop sampling is limited by poor spatial and temporal resolution (Preti et al., 2021). General conclusion on aphid infestation on a wider, regional scale cannot be drawn from individual fields, since aphid infestation levels can vary considerably between fields within a region (Holland et al., 2021).

Aerial sampling, in contrast, relies on flying aphids. These sampling methods can disclose information about the (first) flight activity, the immigration of aphids into and emigration out of the crops as well as the duration of the flight period not only on the local, but also on regional scale (Bell et al., 2015; Harrington and Hullé, 2017). Thus, aphid monitoring by aerial sampling is crucial for monitoring aphid vectors of plant viruses and managing plant virus spread (Krüger and van der Waals, 2020). Here, yellow pan traps and suction traps are the most commonly used autonomous and technically standardized trapping devices to assess aphids in arable crops by farmers, agronomists and scientists.

Pan traps, are (colored) trays filled with liquid that attract and trap flying insects by inducing their landing behavior (Moericke, 1951). An overview for insect monitoring using pan traps has recently been provided by Montgomery et al. (2021). In brief, pan traps are typically placed in open areas, e.g. directly into the crop, with a respective distance to the field margins with the trapping height adjusted to the vegetation level, i.e. initially on the soil between crop plants and later elevated above the crop canopy. Depending on the intended species or group to be monitored, traps of different colors can be utilized. For monitoring aphids, yellow pan traps have shown the greatest trapping efficiency, especially when placed on a high contrast background, e.g. on dark soil (Döring and Chittka, 2007; Döring, 2014). Although selective for certain aphid species (Karl, 1991), over 90 aphid species have been described to be frequently caught in yellow pan traps in Central Europe, among them around 35 species with agricultural relevance (Müller, 1975; Dubnik, 1991; Basky, 1993). Due to their good trapping ability, pan traps are used in a wide range of arable crops, including e.g. sugar beet, legumes and cereals, to assess the initial flight activity and generally monitor migrating aphids on a local scale. Here, vector monitoring in seed potatoes is of particular importance worldwide (Boiteau and Parry, 1985; Harrington et al., 2009; Vučetić et al., 2013; Kim and Kwon, 2019). As an alternative to pan traps, also colored sticky traps can be used to obtain relative measurements of insect populations (Heinz et al., 1992). Instead of being caught in a liquid, here, individuals stick to an adhesive, colored surface of variable size. While catch rates differ depending on traps size and vertical or horizontal orientation (Heathcote, 1957), for some aphid species sticky traps possess better catch rates than pan traps (O’Loughlin, 1963). Since the animals are trapped on the sticky surface, regular visual checks are mandatory to avoid overfull traps where animals overlap. Only this way, an evaluation and identification of the catches is possible.

Suction traps are suitable for regional monitoring, which can be extended to a national and multi-national level (Cavalloro, 1987; Bell et al., 2015; Lagos-Kutz et al., 2020; Ziesche et al., 2020). There are different types of suction traps, with varying technical specifications such as differences in heights, ranging from 1.6 to 12.2 m, leading to a different spatial operating capability (cf. Teulon and Scott, 2006). Even though most of the suction traps present today were installed several decades ago (Macaulay et al., 1988; e.g. Shortall et al., 2009) this approach to monitoring insect pests is far from outdated. In 2001 a dense network of suction traps was established in the Midwest of the USA (Lagos-Kutz et al., 2020) shortly after a first report of the Soybean aphid, *Aphis glycines*, in the year 2000 (Hartman et al., 2001). The suction trap network enabled researchers to observe the rapid spread of *A. glycines* during subsequent years (Ragsdale et al., 2011; Lagos-Kutz et al., 2020). Suction traps have also been used to monitor the spread of the aphid species *Diuraphis noxia* across the USA (Pike et al., 2012).

In Europe, the Rothamsted type suction trap, with a height of 12.2 m, represents the standard trap type for large scale monitoring of migrating aphids in agricultural landscapes, but also collects other flying or drifting arthropod species. Independent of subjective factors, the Rothamsted suction trap can provide technically standardized daily records during the main aphid migration season between April and December (Ziesche et al., 2020). It provides the capability for an absolute population measurement for which insect abundances can be sampled more precisely in comparison to all other methods of aerial catches (Bell et al., 2015). In temperate regions, the life cycle of several aphid species involves host alternation, e.g. between a weed or tree species in winter and an annual crop during summer. Consequently, in these periods, increased numbers of migrating winged aphids can be recorded in suction traps. Additionally, these traps have been proven to provide representative results for monitoring certain aphid populations up to a 30 km perimeter (Starý and Lukášová, 2000). Suction traps conduct sampling with a high degree of temporal resolution over many years and demonstrate their value for arthropod monitoring over large areas (Shortall et al., 2009).

Despite the high potential for agriculture and, for example, the study of climate change effects on species composition, abundance and flight activity of aphids, the operation of a suction trap network requires a lot of work, which is also accompanied by a considerable financial outlay. As a result, networks with a high number of suction traps had to be reduced in size with regard to the number of traps or completely shut down after a few years, as shown for the Soybean Aphid Suction Trap Network in the USA (Lagos-Kutz et al., 2020) and the EURAPHID trap network of the European Community (Cavalloro, 1987). This poses a risk to the collection of long-term data, which is important not only for the study of ecological aspects, but also for the validation, improvement and maintenance of forecasting models.

### 2.2 Identification methods

The disadvantage of all aerial sampling devices mentioned above is their limited selectivity. Besides aphids, a variety of other winged or wind-borne arthropods can be found in such aerial samples. In order to draw any conclusions from the trap catches for a specific target species or group, the taxa of interest must be identified and distinguished from unwanted bycatch.

Traditionally, taxonomic identification of insects is based on different morphological traits. Currently, this is still the method of choice for identifying insects in field monitoring, e.g. when surveying agricultural pest insects. Specimens are identified by eye either directly on the plant, from traps or collected and brought to the laboratory for closer examination. Based on the target taxa, this method of species identification can be very complex, requires a high degree of taxonomic expertise and well-trained personnel. For many insect groups, there are only a few experts worldwide, and the number of well-trained expert taxonomists is constantly decreasing (Engel et al., 2021). Despite the restrictions of this traditional identification method, it still represents a foundation for biodiversity research and integrated pest management programs. When alternative identification methods are developed, the accuracy of these methods should be compared with manual taxonomic identification. These alternative methods should be comparable with, or exceed, the accuracy of standard taxonomic methods. Here, we give a short overview of emerging and promising technologies with focus on the identification of arable pest insects, but not for standardized monitoring, although the listed methods may possess the ability to provide both.

Environmental metabarcoding enables rapid, accurate and cost-effective identification of known species. Taxonomic species identification is performed by means of a species-specific sequence on the mitochondrial cytochrome-c-oxidase I subunit gene (Hebert et al., 2003) and comparing the sequence to a reference database in private or public archives, such as BOLD (Ratnasingham and Hebert, 2007) or GenBank (Benson et al., 2013). Sequences of about eight million insects worldwide have been deposited in BOLD, including nearly 2000 aphid species. Unfortunately, this standardized method only provides presence/absence data or, as recent studies for quantitative barcoding demonstrate, promising results for estimating relative species abundance for (mixed) bulk samples (Liu et al., 2020). Still, metabarcoding does not allow conclusions to be drawn about the exact number of individuals. This is, however, of great interest when assessing insect abundance, including the presence of virus transmitting aphid species, and the severity of immigration flights for pest management actions.

Entomological radar is a remote sensing method that has recently been reviewed by Noskov et al. (2021): With radar, insects can be identified down to species level based on wing beat frequency or size. The identification of larger insects has already been demonstrated a few decades ago (Moore et al., 1986) and mass occurrences of smaller insects including biomass can be detected (Leskinen et al., 2011) but new technologies furthermore show promising results for the differentiation of smaller insects (Wang et al., 2017). Thus, there is a perspective for species and individual identification using entomological radar (Hu et al., 2020). While entomological radar provides the opportunity for a standardized identification and monitoring of flying insects at broad spatial and temporal scales, the identification of many animals in short time intervals, comparable to the catch rate of suction traps, is currently not possible using classical radar technologies. In contrast, entomological laser radar, such as light detection and ranging (LIDAR), enables high sample rates, even for small insects, but currently possess limited identification capabilities (Brydegaard et al., 2021).

Infrared sensors (IR) are capable of detecting flying insect activity by using near-infrared LED lights and high-speed photodetectors (Rydhmer et al., 2022): Different parameters such as the wing beat frequency, melanization and wing to body ratio can be recorded in the field, automatically uploaded to a cloud database, and processed via machine learning (ML) and AI. So, the characteristic morphology of different insect groups can be specified, allowing a remote classification of insects to different taxonomic levels.

Image recognition can be utilized by a wide range of applications related to optic detection of insects enabling remote and non-destructive environmental monitoring (Preti et al., 2021). Previous studies on image recognition of insect pests have been limited to feasibility studies (Wäldchen and Mäder, 2018), and image analysis on natural backgrounds (Cheng et al., 2017; Xia et al., 2018; Patel and Bhatt, 2019). Only a few studies addressed insect identification from mass catches (e.g. Valan et al., 2019). First applications for identifying pests in arable crops using image recognition are close to marketability, e.g. yellow pan trap analysis by Xarvio field manager (BASF Digital Farming GmbH) or MagicScout/MagicTrap (Bayer AG). These apps, however, are often tailored to end users and do not allow species identification at a scientific level with the necessary scientific accuracy. Nevertheless, the fast technological process in the field of image recognition, especially in combination with AI (as explained below), and the possibility of a non-destructive identification makes this method one of the most promising tools of species identification and biodiversity research.

## 3 Image recognition based upon artificial intelligence

### 3.1 Artificial Intelligence methods

In recent years, AI methods have found applications in various fields of science and engineering (Sarker, 2021). The technical possibilities due to increasing computing capacity allow efficient automated evaluations of very large, high-dimensional data such as images and videos. Foremost, deep learning (DL) methods have led to a significant improvement in the quality of results, compared to traditional ML methods (LeCun et al., 2015). In contrast to a manual evaluation of repetitive tasks, such as object recognition and classification, automated solutions offer numerous advantages: they are inexpensive, very efficient, scale well with increasing amounts of data and deliver precise and reproducible decisions. Additionally, various open source implementations allow for a quick empirical data evaluation and comparisons between alternative technical approaches. Programming libraries such as TensorFlow, Keras and PyTorch provide a large number of different models and implementations of various methods (some of which are already pre-trained) from DL with a programming environment for an adaption of tools to the specific application at hand.

As a result, automated DL solutions are widely used in various application areas in biology, agricultural sciences, and ecology (Kamilaris and Prenafeta-Boldú, 2018). Due to the repetitive nature of insect identification, these methods appear to be a suitable tool for the sample analysis of insect mass catches from pan and suction traps (see above).

### 3.2 Machine learning/Image recognition

Many authors, reporting success stories in their disciplines employing AI, use the terms artificial intelligence, machine learning and deep learning almost interchangeably. However, in technical literature, these terms refer to slightly different subject matters (Goodfellow et al., 2016).

AI is the umbrella term for any computer program that can learn from its environment and make decisions based on what it has learned. Specifically, AI encompasses the fields of ML as well as DL, but also includes approaches such as symbolic AI, which relies on a set of given symbolic rules in order to execute its tasks.

ML on the other hand refers to a subset of AI that uses algorithms to learn from data, rather than relying on explicit programming. Based on a set of data, an underlying model is being trained such that the relevant rules for predicting and explaining new data are automatically learned instead of given a priori.

One specific sub-field of ML is DL, which uses a cascade of multiple layers in order to learn increasingly complex representations in the data. The complexity of a representation is defined by the non-linearity and intricacy of the function it can produce given its parameters. DL is based on neural networks, which use a biologically inspired network of interconnected nodes for learning. Each node in the network is connected to other nodes, and the connections between nodes are weighted to represent the strength of the relationship between them. The nodes in the network are organized into layers, with each layer representing a different level of abstraction. The depth of the model is denoted by its number of layers, where in practice, DL networks with up to hundreds of layers are being used.

In ML and DL, model evaluation is typically achieved by dividing the data into a training set and a test set. The model is trained on the training set and its performance is evaluated on the test set. Sometimes, an additional evaluation dataset, known as a validation set, is used during the model development and hyperparameter tuning process in order to assess the model’s performance, while the test dataset is only applied in the final evaluation of the learned model. Here, the idea is to allow for a better estimate of the generalization error of the model by avoiding overfitting to the test set during the learning process.

As evaluation metrics, *accuracy*, i.e. the proportion of correct predictions to total predictions, *precision*, i.e. the proportion of true positive predictions to all positive predictions, and *recall*, i.e. the proportion of true positive predictions to all actual positive instances, are commonly used. These metrics measure the model’s ability to generalize to new data and make accurate predictions.

**Figure 1:**
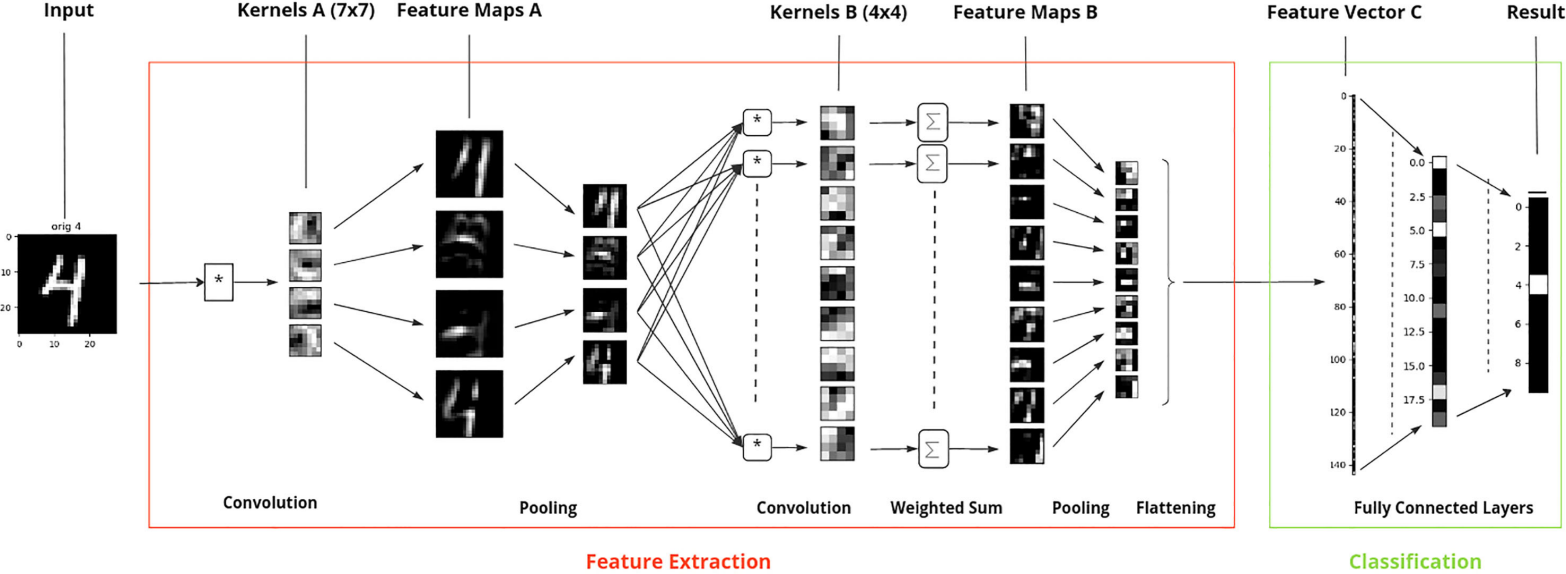
Example of image recognition, here digit identification (range 0 to 9), by convolutional neural networks based on the MNIST database. Starting from left to right, the input image (“4”), the image features are first extracted (red box) and then the image representation based on this feature vector is used for classification (green box). The underlying CNN consists of - six layers, where the first two pairs of convolutional and pooling layers are used for extracting relevant feature information from the image for digit identification and the remaining two layers for classifying the image into one of the ten digit classes. Here, the digit “4” is correctly classified to its true label.

For image recognition applications DL methods are the most common. The most popular type of DL algorithms are Convolutional neural networks (CNNs). CNNs use a combination of convolutional layers, pooling layers, and fully connected layers to identify and classify objects in an image (O’Shea and Nash, 2015). The convolutional layers extract features from an image, such as edges, shapes, and textures, while the pooling layers reduce the size of the feature maps image, while maintaining the relevant information. Fully connected layers, i.e. types of layers that connect every neuron in one layer to every neuron in another layer, are then used to make predictions based on the extracted features. The successive layers start by capturing small scale patterns and progressively move to representations of larger image sections (**Figure 1**).

Recently, so called Vision Transformers (ViTs) have gained popularity in the computer vision community due to their competitive performance to the current state-of-the art CNNs in a number of image classification tasks (Dosovitskiy et al., 2020). While both, CNNs and ViTs, belong to the class of DL methods, they exhibit distinct architectural differences. CNNs process image data in a hierarchical manner, extracting local features that gradually combine to form global representations. In contrast, ViTs divide images into patches, on which a method called self-attention is being applied in order to capture both, local and long-range dependencies, within the patches. By attending to all patches and considering their relationships, ViTs can capture contextual information and understand the global structure of an image. Capturing long-ranging dependencies leads to better contextual understanding and the spatial nature of the self-attention mechanism of ViTs to a better interpretability of results.These abilities offer an advantage of ViTs over CNN models. ViTs are, however, challenging regarding their application as they require a high demand of computational and memory resources as well as larger training sets in order to adequately generalize and overcome sensitivity to variations in input data.

In contrast to DL methods, there are the classic approaches of ML, which employ a data representation by means of only one or two layers. This class of methods such as kernel learning or Bayesian methods (sometimes called shallow learning in the literature in contrast to DL) were the dominant approaches before the breakthrough of DL in recent years (Bishop, 2009).

Usually, when using classical ML methods, the data representation is not learned, but manually annotated instead. Ideally, handcrafted feature selection should transform the original data into a much lower dimensional space while retaining the information necessary for identification. In practice this process turns out to be time consuming and requires deep understanding of the data and the specific task at hand. Hence, the ease of automatically learning data representation in tandem with their good scalability and performance on big data sets have made DL a much more attractive alternative to the classical approaches especially in image recognition tasks.

One way to gain better interpretability and intuitive insight into the learned DL model is by creating synthetic data with the help of Generative Adversarial Networks (GANs) and subsequently comparing the generated images with real world input (Goodfellow et al., 2014). Also, Grad-CAM (Gradient-weighted Class Activation Mapping) is a technique used to provide interpretability in neural networks by visualizing which regions of an image are most important for a given identification or classification task (Selvaraju et al., 2020).

The need for large data sets to train DL models successfully is a well-known problem, especially in applications where additional data is unavailable or can only be gathered with significant costs and efforts. This is frequently the case in challenging image recognition tasks, such as insect identification, which often require hundreds of examples for each species but where specimens of rare species are hard to come by (Borowiec et al., 2022). Fortunately, the use of transfer learning techniques in cases of sparse data availability have achieved surprisingly good results in a number of different applications in practice, even in cases of highly specific image recognition tasks. Instead of learning a new model from scratch, transfer learning is a technique that allows a trained model to leverage the features learned from the original task and use them to improve performance on the new task. This technique is useful when the new task has a similar data distribution to the original task. The weights of the pre-trained model are used as the starting point for the new task and the model is then fine-tuned on the new data to improve its accuracy. In addition to reducing the amount of training data needed for successful training the time required in order to train the model can often be reduced by using transfer learning (Tan et al., 2018; Wang and Deng, 2018; Zhuang et al., 2021).

Image recognition tasks commonly use a number of different transfer learning architectures, with ResNet, Inception, VGG and YOLO being the most popular. Transfer learning models are pre-trained on large datasets such as ImageNet and COCO (Common Objects in Context). COCO is an image recognition dataset designed for object detection, segmentation, and captioning with 330,000 images and 80 object categories, while ImageNet refers to a dataset containing over 14 million images and 1,000 object categories, which include common objects and concepts such as animals, vehicles, and everyday items. The YOLO architecture (Bochkovskiy et al., 2020 *)* was pre-trained on the COCO data, while ResNet (He et al., 2016), Inception *(* Szegedy et al., 2015 *)* and VGG (Simonyan and Zisserman, 2015) used ImageNet for pre-training.

### 3.3 Segmentation, classification and instance segmentation

Most applications in image recognition involve segmentation or classification tasks. Image segmentation refers to the subdivision of an image into multiple segments or regions. This includes both a delimitation of the objects from non-relevant image areas (‘background’) as well as the differentiation of relevant objects from each other (‘instances’). Image classification describes the assignment of an entire image to one of the possible specified classes.

However, in many practical classification scenarios like insect or plant identification, the images to be analyzed frequently contain a number of different instances, each of which must first be identified and isolated in the image before the classification of each individual instance can take place. This combination of image segmentation and classification, which is referred to as instance segmentation in the literature, poses a challenge in many entomological applications: Images from mass trap catches, for instance, usually contain a great number of individuals, comprised not only of different target species but also non relevant by-catch and impurities (Cesaro Júnior et al., 2022), and can possess a high sample density, where individual animals and/or impurities may touch or overlap each other (Borowiec et al., 2022).

There are a number of different approaches to solve the instance segmentation problem- the most popular is Mask R-CNN, a two-stage CNN that first detects and subsequently segments objects in an image (He et al., 2017). The first stage uses a fully convolutional network to generate object proposals and classify those proposals. The second stage generates masks for each object proposal, indicating the area in an image where a relevant object could be located. The masks are then merged, and the resulting segmentation is used to generate the final output.

Several methods are described for the task of classification (Bishop, 2009; Goodfellow et al., 2016). In the context of DL, a softmax classifier, consisting of a fully connected layer followed by a softmax activation function is typically used as the output layer for CNNs as well as ViTs. The softmax function produces a probability distribution over the predefined classes, and the class with the highest probability is considered as the prediction for that input. Further popular choices, used in ML as well as in DL, are support vector machines classifiers, decision trees and random forests, where the latter are an ensemble method of decision trees, combining the results of multiple trees for the final prediction (Wilf et al., 2016; Segev et al., 2017; Wei et al., 2017; Zhang and Zhu, 2018).

### 3.4 Overview: Image classification in entomology

In recent years, there has been a growing interest in applying segmentation and classification techniques to automated insect identification (Martineau et al., 2017; Xia et al., 2018; Zhong et al., 2018). Due to the many challenging tasks of image based expert level identification, fully automatic solutions without the need for manually designed features used in ML only became feasible recently, following the development of DL algorithms. Here, expert level identification means a taxonomic identification of morphologically similar specimen with a high level of accuracy. In many cases, these identification tasks can be challenging even for a human with expert knowledge, e.g. when a species determination is based on small morphological features as in many aphids (cf. Thieme, 1989).

Up to now, publications in the field of insect identification on a species level with a high degree of morphological similarity using AI methods are scarce. In practice, the bottleneck is often the lack of available high quality training data labeled by a human expert, which is, in turn, required for solving complex classification problems. Here, the application of transfer learning methods offers a convenient approach, with good to great performance for a number of different image based classification problems (Borowiec et al., 2022). Next, we will provide a short literature overview of DL based insect identification on data with a high degree of morphological similarity. We note that the approaches discussed below are supervised methods which are unable to detect species that are not included in the training data set. In the field of entomology, very few publications discussing unsupervised techniques for identifying insect instances of an undescribed species exist at the moment. Hitherto, the approaches discussed mainly demonstrate the viability of unsupervised insect identification and are not applicable to real world scenarios, especially with respect to more challenging tasks involving morphological similarity (Badirli et al., 2021).

One recent study was done by Valan et al. (2019), who examined two data sets with a limited number of training data. The first analysis concerns the identification of three closely related species of the Coleoptera genus *Oxythyrea* (339 images), the second example involved nine species of Plecoptera larvae (3,845 images). For both tasks DL based classification achieved better results than manual identification with 97% accuracy on the *Oxythyrea* data set and 98.6% on the Plecoptera data. The images of both datasets were not recorded within a natural setting, but rather possess a monochrome background with slight variations in lighting conditions.

For both experiments, a transfer learning based on the CNN architecture VGG16, a convolutional neural network with 16 layers pre-trained on the ImageNet dataset, in conjunction with a linear support vector machine (SVM) classifier was used.

**Figure 2:**
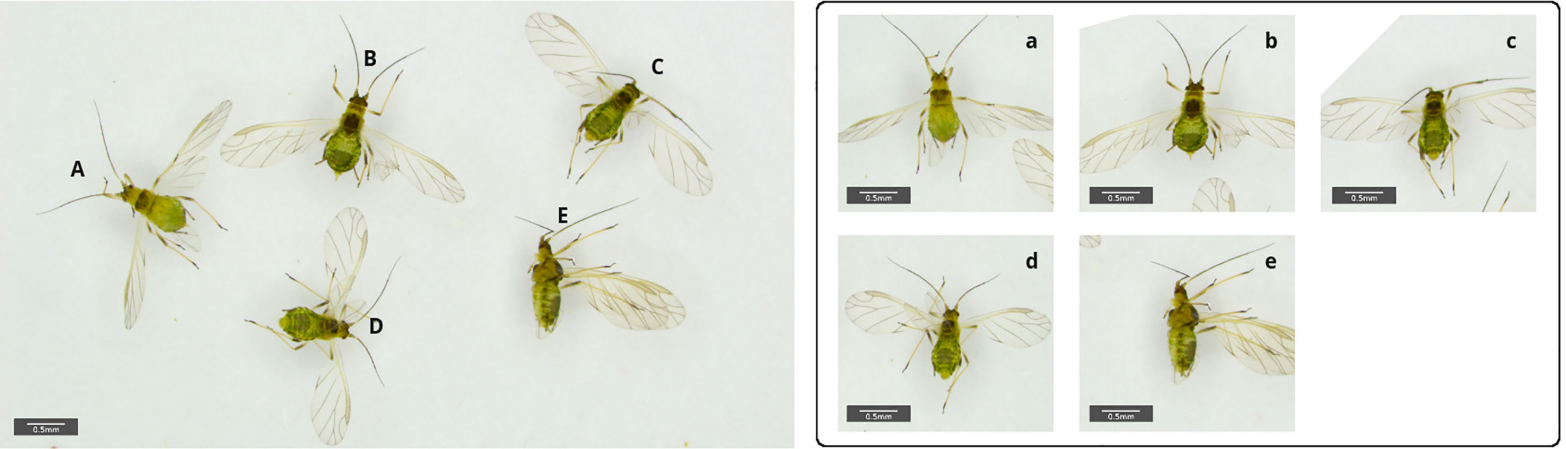
Example for variable orientations of winged morphs (alatae) of the Green peach aphid, *Myzus persicae*, in a sample basin filled with ethanol (70%). The image contains five aphids in different orientation, ventral **(A)**, dorsal **(B–D)** and lateral view **(E)**. Automatic image segmentation and subsequent rotation of the specimens can be used as a first step to simplify further data analysis (right a-e). Pictures are captured with a Leica Emspira 3 digital microscope.

Transfer learning has also been successfully used in challenging identification problems for non-biting midges (Milošević et al., 2020) and adult mosquitos (Motta et al., 2020). Milošević et al. (2020) used a dataset of 1,846 specimens from 10 morphologically very similar species, achieving over 98% accuracy on test data with a CNN architecture ResNet-50, a 50 layered network and therefore significantly deeper than the 16 layer of the VGG16 model used by Valan et al. (2019). Although more complex than VGG16, this model could be reliably trained with under 200 examples per class on average. One reason for the high accuracy is the lab based image acquisition protocol used, which ensured a fixed object position, camera view and angle for each midge from the test data. By closely limiting the variability of the images being used the task complexity implied by the learned feature representation can be significantly reduced, therefore requiring only a modest amount of data. In many applied applications such an intense preprocessing of data for instance in the case of mass trap images is impractical. Instead, one would rather try to learn variable orientations of animals by including relevant images in the training data or alternatively by using preprocessing tools such as automatic rotation and positioning to a reference position (**Figure 2**).

In an additional example, presented by Motta et al. (2020), different transfer learning models were trained to separate mosquitoes from other insects, as well as to classify mosquitoes of genus *Aedes* in comparison to *Culex*. Using a training set of 7,561 mosquito and 1,187 images of beetles, spiders and bees labeled as “other”, identification accuracies of 93% and 97% were achieved. The images used are captured in different resolutions and show the specimens in various positions to the camera together with differing background structure and color distribution. Still, the transfer learning models used in conjunction with an optimization of learning parameters are able to reliably identify the relevant specimens.

The work by Hansen et al. (2020) represents an example of insect identification with a large number of species. Here, an image library of 65,841 museum specimens containing 361 carabid beetle species was used. All images were scanned according to an imaging protocol that defined light intensity, exposure time and orientation of the specimens. By using a pre-trained Inception-v-3 CNN transfer learning approach, accuracies of 51.9% and 74.9% were achieved for species and genus level, respectively, for the test data. Here, the underlying complexity of the problem due to a large variability of many different species together with subtle morphological differences for some cases leads to a significant amount of misclassifications.

A challenging identification task at a genera level was discussed by Marques et al. (2018), where 44,806 ant specimens from the online database AntWeb comprising 57 different ant genera were used for identification. For each specimen, on average 3.35 images in different orientations were available, in particular head, profile and dorsal views, depicting relevant morphological structures for identification. For identification, two different CNN models were trained: one CNN over all images and views and one ensemble of three CNNs with one neural network for each of the three orientations together with a classifier combining the three answers. In the experiments, the ensemble model (accuracy > 90%) significantly outperforms the standard model (accuracy > 80%), showing that separation into distinct sub-models helps to better preserve morphological information relevant for identification, which in turn leads to better classification.

Nanni et al. (2022) presented an empirical comparison using different transfer learning architectures on three benchmark pest data sets Deng, D0 and IP102. The data sets range from 563 images divided into ten insect classes (Deng) over 4,508 images divided in 40 insect classes (D0) and 75,222 images divided into 102 classes of pests categorized into a hierarchical taxonomy (IP102). Classification of pest data is usually complex, since images collected in the field contain not only the relevant pest objects but also the surrounding environment. Here, the object often constitutes only a small portion of the image. Therefore, fine grained analysis is necessary to differentiate the insect classes, while variability in the images is often high due to variable environmental conditions. As CNNs in this study, six different models (ResNet50, GoogleNet, ShuffleNet, MobileNetv2, DenseNet201, and EfficientNetB0) are being used, where these nets are being evaluated as combinations of models in an ensemble. For the optimization, the authors apply different versions of Adam, a variant of stochastic gradient descent methods. The results show that ensemble methods increase the accuracy in comparison to stand-alone CNNs, with 95.52% accuracy compared to 94.64% (ResNet50) on Deng and 74.11% compared to 73.12% (EfficientNetB0) on IP102. For the D0, an accuracy for the ensemble method of 99.81% is reported. For the Deng dataset a study of six human experts shows accuracy rates between 82% and 96%, so the model exhibits comparable accuracy rates to the most accomplished human experts.

The potential of ViT models in pest classification have been evaluated by Xia et al. (2022). The authors use ResNet50, MMAINet, DNVT and an ensemble learner combining the predictions of these three models in a final classification vote. MMAINet uses an attention mechanism identifying discriminatory image regions, on which fine grained CNN based classification models in different resolutions are being trained. DNVT is a hybrid architecture that combines a DenseNet CNN with the self-attention mechanisms of ViTs, which enables effective feature extraction together global context modeling. The empirical part reports results from the two pest datasets D0 and IP102. On both data sets, the best accuracy is achieved by the ensemble method (99.89% on D0 and 74.20% on IP102). However, a comparison of the accuracy for the individual models ResNet50, MMAINet and DNVT shows that the ResNet50 model performs best for both data sets (99.37% on DO and 71.71% on IP102). Here, the decision between the ensemble method and the ResNet50 for the user is one of accuracy versus computational efficiency, since the latter is computationally significantly less costly than the ensemble method.

Also Liu H. et al. (2022) propose a ViT classification model, which uses two preprocessing techniques for performance improvement. Since ViT models require a substantial amount of training data which cannot be provided by pest data sets, the authors employ a pre-training method to generate suitable training data and subsequently learn discriminative features. Specifically, a FRCF algorithm filters from the ImageNet dataset commonly used in transfer learning techniques a relevant subset, which is similar to the pest data to be classified. Then a ViT based LSMAE model is trained, which extracts a discriminatory feature representation from the semantic information in the image patches. The pest datasets are then used to fine tune the classifier. The evaluation is based on pest data sets IP102, CPB and PlantVillage, where CPB contains 10,816 images of six different mite species and a class of non-mite, while PlantVillage is a plant dataset containing 54,305 images from 14 plant species. The results report state-of-the-art accuracy on all three datasets 74.69% on IP102, 76.99% on CPB and 99.93% on PlantVillage.

In all cases of expert level classification models, deep transfer learning performs superior in terms of prediction accuracy compared to reference study models, indicating that this is the current state of the art approach for image classification in entomology, including aphid identification. The choice of the best model for a certain problem depends on several factors, in particular the number of images of individuals and the image quality of the training dataset, as well as the complexity of the underlying identification problem. The availability of a large number of transfer learning models as open-source software implementations allows for a relatively simple empirical comparison of different approaches.

### 3.5 Overview: Applications towards aphid identification

Several studies address the identification and classification of aphids. For each of these, the complexity of the respective task varies considerably and depends on factors such as image quality, sample purity and complexity of the samples. The challenges are determined by various factors:

1. Image capturing conditions: Is it possible to create a training set under standardized settings (exposure, image quality, background, body orientation) or does variability have to be created? This requires information from the sample and the future imaging conditions of the samples to be analyzed.
2. Image composition: What sample density is expected for the test set? Animals in dense samples may touch or even overlap each other. When arthropods other than aphids appear in a sample, as is the case for pan and suction trap catches, other taxonomic groups must be considered for classification.
3. Classification: Should aphids only be counted or should a distinction be made into species or species “types”, respectively? How many species should be distinguished, how big are the morphological differences and furthermore, are specimens present in different morphs and developmental stages (nymphs, wingless, winged) and states of conservation? In case that samples from pan and suction traps are analyzed, only winged adult aphids are expected.

### 3.6 Classical machine learning

Xuesong et al. (2017) used data from sticky boards as a basis for identifying and counting aphids. Statistical methods of classical ML were used as recognition algorithms. Here accuracies of over 90% are reported for both, in the greenhouse and outdoors, where the accuracy in the field experiments turned out to be slightly lower due to differences in diurnal lighting conditions. A distinction between aphids and other insect taxa was based on a simple size measurement, which, however, will not enable a robust differentiation in many practical applications, since several Hymenopteran and Dipteran species possess a comparable body size to aphids.

### 3.7 Deep learning

A method to analyze and classify populations of *R. padi*, using DL methods was developed by Lins et al. (2020). By segmenting 30,000 individual specimens, the model is capable to distinguish three different developmental stages (nymphs, winged adults, unwinged adults) as well as a differentiation of impurities. Standard image recognition methods were used for segmentation from the sample images, while the classification of the segmented specimens was carried out using CNN Inception-V3. A comparison with a manually performed classification shows a better recognition rate for all three aphid classes, which is quantified by the number of specimens found. The solution of a relatively simple classification problem based on large data processed in the laboratory can thus be readily solved using already available DL procedures.

Another approach is discussed by Hayashi et al. (2019), where the neural architecture search (NAS) tool of Google AutoML Vision is used to identify three aphid species (*Aphis craccivora, A. pisum, Megoura crassicauda*) from plant images. In order to evaluate the influence of the size of the training dataset, different models with a fixed number of training images per species, ranging from 20 to 400 images, were trained. One hundred training images per species were required for an accuracy of over 90% and with 400 images per species an accuracy of over 96% was achieved. However, since the species used in this study are easy to distinguish by the non expert in terms of size and color, the model does not capture the complexities of identifying morphologically similar aphid species.

The presence of aphids on images taken from lemon tree plants in the field, which feature natural background variability and changes in lighting conditions, was solved by Parraga-Alava et al. (2021).To do so, 150 images of plants were taken as training data (70 images of healthy plants, 80 images of plants infested with aphids) and a transfer learning approach with a VGG-16 network architecture was used. The authors report a classification of infested and uninfested plants with rates between 81% and 97% on lemon tree plant images of the test data set. While the objective in this experiment only involved detection of aphids, the variability induced by images taken under changing conditions in conjunction with a small sized training data set made the task more challenging.

Regarding network architecture, an almost comparable approach was used for segmentation and counting of *M. persicae* nymphs and a classification of nymphs and adults on leaves using a transfer learning system with a VGG-13 architecture (Chen et al., 2018). Here, 68 images of different plant leaves, with up to a hundred nymphs per leaf, were used for model training. In the evaluation of the aphid count on the corresponding test images, the model achieved a precision of 95.6% - and a recall of 96.5% respectively. The sample complexity can be considered as low, with no by-catches and only one aphid species and just a distinction into nymphs and adult aphids.

These studies can be regarded as interesting applications for aphid detection adapted either to a specific type of application or to a simplified modeling environment. Hence, their direct applicability in more complex practical scenarios of a wider scope involving many different aphid species of high morphological similarity seems rather limited.

### 3.8 Deep learning on images from mass trap catches

Cesaro Júnior et al. (2022) introduced a system for insect detection from actual field trap images (yellow pan traps), and studied the performance of an AI approach for samples of different insect density with the aim to develop an online AI tool for integrated pest management. Sample images were complex, containing hundreds of winged insects, including aphids, parasitoids, flies and thrips, all in different orientations and/or partly overlapping. Additional segmentation and identification challenges included the occurrence of contamination (dust and other small particles) as well as specimens in various states of conservation. The latter in particular seems to be of great importance for the analysis of images: While training sets often consist of intact and freshly prepared animals to perform initial learning under optimal conditions, samples from field collections may contain a considerable amount of insect fragments and contaminants. Additionally, trapping aphids in conservation liquid under field conditions can also lead to color change, and with regard to gray-scale images as used by Cesaro Júnior et al. (2022) to changing gray values, but also to a change of body shape due to inflation. The authors used a dataset of 17,908 labeled insects, comprising 9,783 aphids and 8,125 parasitoids. The automated identification and counting of the aphid and parasitoid populations was carried out with a Mask R-CNN algorithm.

To evaluate the method, the computed numbers of aphids and parasitoids were compared with the manual counts of a human expert. Here, equivalent values for precision (85%) and recall (41%) were determined for the examined test images for parasitoids and aphids. Thus, on average, significantly less than half of the relevant specimens (determined by manual counting) could be identified. This shows that the proportion of discovered specimens decreases noticeably with increasing density of animals in the image. As reasons, the authors state the high degree of contamination, the morphological similarity of the bycatch and the target aphids for identification as well as the pose variations. In practice this means that a dilution of the sample could be necessary in samples comprising a high insect density.

Although state-of-the-art methods are used here, the performance is significantly lower than that of comparable entomological studies dealing with insect counting. The authors attribute this to the complications of pollution, (partial) overlapping of insect bodies and the presence of by-catch as listed above. These interfering factors did not occur in the data sets of the other studies described. This once again illustrates the challenges that have to be taken into account, especially with regard to the acquisition and processing of the images to be examined in an adequate manner in order to facilitate good identification results. The work of Cesaro Júnior et al. (2022) illustrates, that a system that aims to perfectly assign the segmented images to the respective species falls short of a manual evaluation at the current time if a significant proportion of the aphids are not separated from the by-catch beforehand as part of sample processing.

A table with all publicly available datasets discussed in sections 3.4 - 3.8 is provided (**Table 1**).

**Table 1:** Publicly available datasets from different publications that have been discussed in sections 3.4 - 3.8.

Authors	Year	Title	Link
Valan, M., Makonyi, K., Maki, A., Vondráček, D., and Ronquist, F. (Valan et al., 2019)	2019	Automated taxonomic identification of insects with expert-level accuracy using effective feature transfer from convolutional networks	https://datadryad.org/stash/dataset/doi:10.5061/dryad.20ch6p5
Hansen, O. L. P., Svenning, J.-C., Olsen, K., Dupont, S., Garner, B. H., Iosifidis, A., et al. (Hansen et al., 2020)	2020	Species-level image classification with convolutional neural network enables insect identification from habitus images	https://zenodo.org/record/3549369
Marques, A. C. R., M Raimundo, M., B Cavalheiro, E. M., F P Salles, L., Lyra, C., and J Von Zuben, F. (Marques et al., 2018)	2018	Ant genera identification using an ensemble of convolutional neural networks	https://zenodo.org/record/1134690
Wu, X., Zhan, C., Lai, Y., Cheng, M., & Yang, J.	2019	Insect Pest Dataset (IP102)	https://github.com/xpwu95/IP102
Bollis, E., Pedrini, H., & Avila, S.	2020	Citrus Pest Benchmark (CPD)	https://github.com/edsonbollis/Citrus-Pest-Benchmark
Hughes, D.P., & Salathé, M.	2015	Plant Village Dataset	https://www.kaggle.com/datasets/emmarex/plantdisease
Xie, C., Wang, R., Zhang, J., Chen, P., Dong, W., Li, R., Chen, T., & Chen, H.	2018	Xie2 (D0) Dataset	http://www.dlearningapp.com/web/DLFautoinsects.htm

### 3.9 Difference in testing protocols

The papers discussed vary significantly in terms of test setup and data type. Theoretical studies typically assess methods using benchmark datasets, while applied studies address the challenges of practical implementation. These challenges encompass diverse aspects, such as integrating different data sources, like cloud-based solutions, accounting for variable weather conditions affecting field recordings, and managing bycatch and contamination in large-scale captures. Consequently, the test protocols in these experiments are tailored to address specific questions. For instance, they may involve preselecting training and test images or adjusting recording conditions, which distinguishes them from the typically randomized test protocols used in theoretical investigations. Consequently, despite sharing common target values, such as accuracy, the direct comparability of results is often limited in practice.

### 3.10 Towards expert level identification of aphid species by AI as basis for an automated agricultural pest monitoring (limitations and constraints)

Based on previous studies, the most promising tools for expert level identification of agricultural relevant aphid species by image recognition can be found in the field of DL with the use of CNNs. However, the development of an automated system to record, segment and classify aphids from mass trap catches faces a number of different challenges:

- The sampling method must be suitable for image recognition. Sticky traps, for instance, can have the disadvantage that specimen are partly covered by glue remnants, bycatch or other airborne particles. It is important that the sampling method or sample preparation are adjusted to the needs of ideal image recording and subsequent AI analysis.
- The recorded sample images must be segmented into individual specimens; individuals other than aphids as well as any contamination has to be detected and filtered out during AI analysis.
- Individual specimen are recorded in different orientations to the camera (caudal, lateral, dorsal, ventral, cranial) and may be in contact or overlapping with other insect bodies.
- Depending on the sampling intervals (emptying trap containers) and temperature conditions, aphid individuals may occur in different conservation stages, i.e. the longer aphids stay in the trapping solution the higher the degree of color change and the change of body shape (bloating) in contrast to freshly caught aphids. Furthermore, wax deposits on the cuticule, e.g. typical for different aphid species, may disappear. Regarding the described effects, significant intraspecific differences may appear, with strong effects for some species while for others only slight morphological changes are expected depending e.g. on the color of a species and the melanization of cuticular components. On a more abstract level, this corresponds to the challenging class of classification problems with a high intra-class variance and small inter-class variance. Consequently, these kind of problems require classification methods that are able to model a high degree of complexity, which in turn require a larger number of relevant training examples for reliable and robust classification results.
- Potential morphological difference in-between aphid species must be ruled out or accounted for, when collecting training data.
- Aphid species abundance and species composition can show strong regional variation, possibly also leading to the presence of aphid species with exotic host plants depending upon the location of a trap and the surrounding plant community (Bell et al., 2015). In addition, a spatio-temporal morphological variability has to be taken into account in the training data, as shown for seasonal variation in *M. persicae* (Taylor and Robert, 1984).
- To avoid overfitting of strongly represented classes, it is mandatory to homogenize the training datasets of the classifier for each species.
- High-quality sample data validated by human experts is essential for a precise training of the algorithm. A focus on economically important pest species may help to reduce the complexity of a training data set as rearing of animals in the greenhouse may improve its quality due to a high number of images of the training data set.

Hitherto, to the best of our knowledge, no study presented an adequate solution for AI based expert level aphid identification from mass catches. However, promising models in the broader field of insect identification, demonstrating remarkable results for a variety of different insect species, could be applicable to this specific context.

## 4 Modeling and forecasting

### 4.1 State of aphid forecasting

In agriculture, monitoring of, and forecasting models for pest insects, are by their nature related, as forecasting models almost always rely, at least partly, on data derived from monitoring activities. Monitoring and forecasting are conducted and developed, respectively, with the aim to ensure crop protection by continuous improvement of cultivation methods. Measures may include the adjustment of the sowing time to minimize aphid infestation at early crop stages, to plan crop rotation to control pest emergence or diseases spread, and finding the right timing for plant protection measures such as the application of insecticides (Dedryver et al., 2010) in the sense of integrated pest management. This makes it even more important to time the use of crop protection products according to pest infestation. Here, forecasting models can aid the decision making process.

To establish a forecasting model for an insect pest, knowledge about the biology and life history traits of the pest species is mandatory. Additionally, a basic understanding of the development of insect pests in context with environmental conditions, such as weather or climate, is vitally important for the development and improvement of such models. Here long-term monitoring networks, such as the suction trap networks (e.g. Tilman et al., 2006) are designed to study the impact of climatic or environmental changes on population dynamics and effects on the diversity of species on a spatio-temporal scale (Bell et al., 2015).

In the last forty to fifty years, several aphid forecasting models have been developed using a wide variety of modelling approaches, with the aim being to predict the occurrence and population dynamics of different aphid species in arable crops. A table listing the most important studies and aphid models for different crops is included in the **Supplementary Material** (SM1). A number of the existing models focus on the phenology of a herbivore and its host in relation to abiotic factors, which has proven to achieve significant prediction at a regional level by using monitoring data (Harrington and Hullé, 2017). In addition, models that can predict the migratory behavior of aphids, including arrival time, are of significant interest as these models will identify periods with high migration risk where in-field monitoring efforts could be targeted. Furthermore, as soon as certain aphid pest species occur in e.g. suction or pan traps, subsequent field observations might be necessary to assess aphid abundance on the plant level.

For the holistic evaluation of cropping systems and the further development of alternative crop production strategies, generally model-based methods are used. For aphid pests, these are generally invasion warnings when winged aphids start flying from their overwintering hosts into crops or, with warming winters, when they increasingly survive in the field. Modeling approaches have been developed for numerous important crops and pests such as the simulation model “GTLAUS01”, which modes the population dynamics of three important aphid species in cereals (Gosselke et al., 2001), or “SIMLAUS”, which allows predictions either for a specific crop or for a specific region and aims at forecasting possible outbreaks of BYDV by calculating the probabilities of reproduction and survival of three cereal aphid species (Klueken et al., 2009).

As a practice applied prediction method, near real-time pest incidence data coupled with remote sensing data and GIS tools facilitate early warning of impending pest build-up in space-time resolution, whereat the measure of weather data from pest-affected areas is still an essential input for models (Prasad and Prabhakar, 2012). As insect development is mainly influenced by weather factors, temperature and precipitation data are directly tied to the success of predicting population dynamics in a particular region (Dent and Binks, 2020).

Forecast models are available for potatoes predicting the spread of a Potato virus Y in the current season using trap data of aphid flights (Steinger et al., 2015) to optimize virus control in seed potato production. Qi et al. (2004) review the procedure and decision making process for controlling *M. persicae* and predicting outbreaks of virus yellows disease in sugar beet in the UK. While surveys began in 1946 and a first virus yellows spray warning scheme was introduced in 1959, the forecasting model has been continuously developed since.

Models often simulate one or a few species and rely on the most complete information possible on the auto-ecological demands of the developmental stages which respond to the prevailing environmental conditions. Still, successful forecasting techniques are those that are as simple as possible and that are based on very precise knowledge of the biology and ecology of the pests concerned (Prasad and Prabhakar, 2012), such as the first flight activity or the survival rate of overwintering aphids in temperate regions.

Thus, forecast models are used as decision-making aids to optimize the temporal and spatial planning of infestation surveys, to estimate the need for control and the scheduling of control measures (Dedryver et al., 2010). Effective decision support tools are required in order to provide agricultural practitioners with advice regarding appropriate and economic pest management strategies (Duffy et al., 2017) and to complement recent changes regarding pesticide regulations in the European Union aimed at a general reduction of pesticide applications (Lechenet et al., 2017).

In practice, however, successful decision making depends upon the availability of integrated, high quality information (Harrington and Hullé, 2017) and the information-base should be ensured continuously and in high resolution by extensive monitoring. Generally and with changing conditions during climate change in particular, forecast models of aphids need to be compared and validated regularly for a wide-scale use in crop protection (Klueken et al., 2009; Zeng et al., 2020).

### 4.2 Utilization of AI in aphid forecasting

AI could be utilized to assist and enhance aphid pest forecasting in several ways, by 1) automated identification of insects, whether from suction/pan traps or camera-equipped traps, based on image recognition and DL, 2) development of new forecasting models based on ML or neural networks (e.g. Jarošová et al., 2019), and 3) optimizing the monitoring infrastructure to improve predictive models (Bourhis et al., 2021). Due to the objective of this review, we take a closer look at the potential of AI based, automated identification based on image recognition.

Almost all current models use different aspects of the occurrence or abundance of aphid pests in correlation with weather data (SM1). While abiotic factors, such as temperature or precipitation, can be assessed easily over a large spatial scale, and are often provided by meteorological services, the spatial resolution of monitoring locations is often limited. Monitoring networks, with a representative number of monitoring locations (e.g. traps), are often missing, although highly desirable, because to gain robustness in forecasting accuracy, it is mandatory to capture significant spatio-temporal variations in pest insect abundance in suitable numbers over multiple seasons, so subsequent generalized predictions by forecasting models are meaningful (Bourhis et al., 2021). In turn, operating a monitoring network requires at least some sort of pest insect identification, as explained before. This is often the limiting factor for spatially explicit monitoring.

Here, an AI-based automated identification of aphid pests from mass trap catches, but also other traps that require an image based identification, such as camera-equipped traps using image recognition, has a high potential to overcome the current limitations in monitoring insect pests. Benefits are: 1) the handling time (sorting, identification) of catches could be reduced, allowing large catch volumes to be processed in relatively short time and data to be available on a daily basis, 2) the identification is independent of a human expert, with standardized results, as individual person error is eliminated, 3) an AI based identification application can be used at multiple sites or as a cloud application, thus enabling standardized monitoring of aphids within a large monitoring network with high spatio-temporal resolution, 4) thanks to prompt sample processing, invasive insect pests and associated plant diseases can be detected more quickly with a higher success rate.

This way, future forecasting models could be set on a profound basis. Monitoring data, such as aphid flight data, collected over a wide area enables the creation and verification of more accurate models (Bell et al., 2019). This facilitates complex analyses including the interaction of climate change, land use, cultivation methods, and the occurrence of insect pests, but also allows for the optimization of cultivation systems with regard to pest infestation and virus transmission or the influence of global warming on the abundance and distribution of insect pests. In the long term, this would allow for a continuous assessment of the pest potential of individual species.

That these goals are already within reach is shown by a recent study concerning insect pests in cotton fields in China. Liu C. et al. (2022) developed an autonomous pest monitoring and forecasting device that is capable of capturing and transmitting images of phototactic insects caught in a modified light trap. The insect images are subsequently identified on a local server by methods of DL algorithms. Unfortunately, details on species identification were not yet addressed by the authors, since their main focus was to compare different DL methods for background removal to optimize the image for classification. Nevertheless, it clearly demonstrates the potential of automated, AI-based monitoring and outlines the current state of the art.

## 5 Conclusion and prospects

Information on insect occurrence serves a number of purposes, including research on current scientific questions about changes in biodiversity and species abundance. But also the implementation of important and more applied agricultural tasks such as monitoring the spread of (new) vector-transmitted plant diseases, reducing the use of chemical pesticides as part of the European Green Deal, and the early development of resistant crops in case of the emergence of new pest insects, relies on a profound understanding of the distribution and abundance of insects. This knowledge, however, requires a great amount of insect related data with an adequate temporal and spatial resolution, as was planned to acquire, for example, within the EURAPHIS network.

An automated identification of insect pests, supported by AI as outlined above, could be a promising tool to enable a timely processing of a large number of samples from mass trap catches, which subsequently can lead to timely and highly specific insect pest warnings. The decoupling of the identification process from a human expert and the associated relatively low costs for operation and data analysis could form the basis for comprehensive long-term monitoring activities, not only in Europe, but also in other regions. This form of monitoring is already used at a regional level for biodiversity research. At least for biodiversity research, the importance of such has already been clearly demonstrated at regional level (e.g. Seibold et al., 2019).

The provision of data from mass trap catches in cloud-based databases would allow a large number of research groups to utilize and evaluate the data for various research questions, such as the adaptation of forecast models in connection with climate data and data on regional crop production by AI-based models.

Research activities in image based identification, but also other identification disciplines, will be essential for future biodiversity, and consequently pest insect monitoring.

## Author contributions

CJ and TW conceived the idea of this review. PB, TW and CJ wrote the original draft of the manuscript. TZ and ST wrote sections of this manuscript. PB, TW, CJ critically edited the manuscript. ST prepared and revised the figures. All authors contributed to manuscript revision, read, and approved the submitted version. All authors contributed to the article and approved the submitted version.
