# Supplementary material for: From identification to forecasting: the potential of image recognition and artificial intelligence for aphid pest monitoring
Source: Front Plant Sci. 2023 Jul 19;14:1150748. doi: 10.3389/fpls.2023.1150748 (PMC10396399; doi:10.3389/fpls.2023.1150748)
Supplement: Supplementary file 1 [file DataSheet_1.pdf]

Supplementary Material

From identification to forecasting: The potential of image recognition and artificial intelligence for aphid pests monitoring

Philipp Batz, Torsten Will*, Sebastian Thiel, Tim Mark Ziesche, Christoph Joachim
*** Correspondence:** Corresponding Author: torsten.will@julius-kuehn.de

SM1: Relevant literature on aphid modeling, forecasting and population simulation sorted by affected crop and year, repsectively. Literature without a connection to pest insect forecasting and/or agricultural relevance were not considered.

Crop	Country	Aphid species	Aim / scope	Year	Stated purpose	Reference
			**Brassicas**			
Kale	Australia	*Brevicoryne brassicae*	aphid population dynamics	1968	"[T]ool to understand the combined effects of species interactions and environmental factors on population growth"	Hughes and Gilbert (1968)
Kale	Canada	*Brevicoryne brassicae*	aphid population dynamics	1984	"A computer model was written to simulate the population dynamics of the cabbage aphid, *Brevicoryne brassicae* (L.), on the host Maris Kestrel kale, *Brassica oleracea* L., at Vancouver, British Columbia."	Raworth (1984)
Kale, cabbage, cauliflower, broccoli	Ethiopia	*Brevicoryne brassicae*	aphid population dynamics	2020	"This study provided information on time of initial infestation and also the peak activity of the particular insect in relation to temperature."	Shonga and Getu (2021)
Mustard	India	*Lipaphis erysimi*	aphid population dynamics, first aphid emergence	2005	"Hence, the present study was undertaken to develop forecasts for crop age at time of attack by *L. erysimi* , peak number of aphids on the crop in the season and crop age at peak population of the aphid."	Chattopadhyay et al. (2005)
Mustard	India	*Lipaphis erysimi*	Revision previous model, aphid population dynamics	2008	"This paper extends the previous cumulative-size dependent models to include immigration."	J. H. Matis et al. (2008)
Mustard	India	*Lipaphis erysimi*	aphid population dynamics	2014	"Experimental data from six north Indian locations were used to study the role of weather on the incidence and development of mustard aphid."	Rao et al. (2014)
Mustard	India	*Lipaphis erysimi*	aphid population dynamics	2020	"In this study, an effort is made to develop forewarning model using the field data on aphid for 12 consecutive rabi seasons from 2003–2004 to 2014– 2015 under different agro-climatic locations in India."	Tharranum et al. (2020)
Oilseed rape	Australia	*Myzus persicae*	Beet western yellows virus (BWYV) incidence, aphid population dynamics	2010	"The aims of this study were to: (i) adapt the hybrid mechanistic/statistical model of Maling et al. (2008) to predict aphid vector activity and epidemics of BWYV spreading from a nearby external source to a *B. napus* crop […]."	Maling et al. (2010)
Oilseed rape	Iran	*Brevicoryne brassicae*	50% aphid emergence	2016	"The aim of the study was to develop a model based on degree-days for predicting 50% emergence of the cabbage aphid population in canola fields."	Nematollahi et al. (2016)
Oilseed rape	Australia	*Myzus persicae*	aphid population dynamics	2021	"Here, we develop a mechanistic model to explore the population dynamics of *M. persicae* and *D. rapae* during the canola growing season in southern Australia."	Barton et al. (2021)
Rapeseed mustard	India	*Lipaphis erysimi*	aphid population dynamics	2012	"Thus, an attempt has been made here to predict the appearance and development of aphids on mustard crop for the eastern plains of Rajasthan agro-climatic conditions which ultimately may culminate into a Decision Support System (DSS) for the management of aphids."	Rao et al. (2012)
Rapeseed mustard	India	*Lipaphis erysimi*	aphid population dynamics	2017	"The weekly aphid population on timely sown mustard crop and daily weather parameters […] were used to develop forewarning model for aphid."	Tharranum et al. (2017)
			**Cereals**			
Cereals	England, Netherland	*Sitobion avenae*	aphid population dynamics	1980	"Two models simulating the population development of *Sitobion avenae* are described."	Carter and Rabbinge (1980)
Cereals	France	*Sitobion avenae*		1984	"A very simple linear multiple regression model has been ddevised which is based upon seven years (1975-1981) of samples and catches and on related climatic data: […]"	Pierre and Dedryver (1984)

Crop	Country	Aphid species	Aim / scope	Year	Stated purpose	Reference
**Cereals (continued)**						
Cereals	Finland	*Rhopalosiphum padi*	summer population size	1989	"The present forecasting method which has been taken into preliminary use is based on monitoring the overwintering population […], migration […] and on the estimation of wingless aphi generation on cereal. This report reviews the preliminary use of the above forecasting method and presents an evaluation of its applicatility for predicting the *R. padi* outbreak 1988."	Kurppa (1989)
Cereals	Germany	*Sitobion avenae, Rhopalosiphum padi, Metopolophium dirhodum*	aphid population dynamics	2001	"[The] GTLAUS tri-trophic model for simulation wheat–aphid–antagonist interaction developed by Freier et al. (1996) has been revised with the aim of improving the model details for better modelling of antagonist effects, etc. This paper describes the latest version, GET-LAUS01, and documents the results of model validation and predator effect simulations using field data collected over 8 years."	Gosselke et al. (2001)
Cereals	France	*Rhopalosiphum padi*	Barley yellow dwarf virus (BYDW) incidence	2003	"Logistic regression was used to examine whether a simple risk probability algorithm based only on the autumnal population dynamics of *R. padi* can accurately predict yield losses caused by BYDV and, therefore, the need for insecticide treatment."	Fabre et al. (2003)
Cereals	France	*Rhopalosiphum padi*	Barley yellow dwarf virus (BYDW) risk	2006	"The purpose of this paper is to exemplify this powerful approach through its application to the design of a user-friendly model based on a temperature dependent simulation of *R. padi* population dynamics during autumn in cereal fields to assess the risk of BYD epidemics."	Fabre et al. (2006)
Cereals	Canada	Cereal aphids	future aphid distribution	2006	"In the present paper, I provide projections for aphid population changes in the last quarter of this century for the much larger area comprising the wheat-growing regions of Southern Canada […]."	Newman (2006)
Cereals	UK	*Rhopalosiphum padi*	aphid population dynamics	2006	"A spatially explicit individual-based simulation model has been developed to represent aphid population dynamics in agricultural landscapes."	Parry et al. (2006)
Cereals	New Zealand	*Rhopalosiphum padi*	aphid abundance	2008	"An ensemble model based on artificial neural networks (ANN) was developed to predict the number of *Rhopalosiphum padi* (L.) (Homoptera: Aphididae) caught in traps during the autumn flight period at Lincoln, Canterbury, New Zealand, over the period 1982–2003."	Lankin-Vega et al. (2008)
Cereals	Australia	*Rhopalosiphum padi*	Barley yellow dwarf virus (BYDW) incidence	2009	"BYDV PREDICTOR, a simulation model, was developed to forecast aphid outbreaks and Barley yellow dwarf virus (BYDV) epidemics in wheat crops in the grainbelt region of southwest Australia […]."	Thackray et al. (2009)
Cereals	China	*Sitobion avenae, Schizaphis graminum, Rhopalosiphum padi*	aphid population dynamics	2013	"This project is aimed to develop a detailed aphid population dynamics analysis and forecasting system on three control factors: weather, crop and natural enemy, based on the swallowtail catastrophe model and recent advances in computer programming technologies."	Piyaratne et al. (2013)
Cereals	UK	*Sitobion avenae*	population size / timing	2017	"This work describes a new simulation model that quantifies the size and timing of grain aphid populations in response to temperature."	Duffy et al. (2017)
Cereals	Czech Republic	*Sitobion avenae, Metapolophium dirhodum, Rhopalosiphum padi*	aphid abundance in winter wheat	2019	"In this paper, we used the 15-year data on the flight activity of cereal aphid species [...] and the data of aphid abundance in the plots of winter wheat [...]. We addressed four issues: (i) the seasonal patterns of the flight activity in the particular species as revealed by the suction trap catches, (ii) comparing the species composition of the aphid populations in the suction trap catches and in the winter wheat stands, (iii) establishing the relationship between the aphid abundance on the winter wheat stands on the ensuing size of the suction trap catches and (iv) predicting the abundance in the winter wheat stands from the suction trap catches."	Honěk et al. (2019)
Cereals	China	Cereal aphids	aphid population dynamics	2020	"In this paper, first, a wheat aphid population dynamics model was developed based on a logistic model and the Holling III functional response, which includes three factors: temperature, natural enemies and insecticide. Second, this model fitted with a cusp catastrophe model to describe how abrupt changes in the wheat aphid population were influenced by these factors."	Li et al. (2020b)

Crop	Country	Aphid species	Aim / scope	Year	Stated purpose	Reference
**Cereals (continued)**						
Barley	New Zealand	*Cereal aphids*	Barley yellow dwarf virus (BYDW) incidence	1999	"In the present paper, we describe a year, 1998, of high BYDV incidence in autumn wheat in Canterbury, examine its probable causes and discuss its implications to the aphid monitoring-virus forecasting programme."	Teulon et al. (1999)
Barley	North America	*Diuraphis noxia*	aphid population dynamics	2008	"In this paper, we focus on the application of survival analysis to simulation of insect population dynamics, again, with [the Russian wheat aphid, *Diuraphis noxia*] population as the example."	Ma and Bechinski (2008)
Barley	Germany	*Sitobion avenae, Metapolophium dirhodum, Rhopalosiphum padi*	fall and spring migration	2009	"Accordingly, the aim of our investigations was to determine relationships between mostly meteorological parameters and cereal aphid flight activity in order to identify reliable predictors of aphid immigration into winter wheat and winter barley crops in autumn and spring for modelling purposes."	Klueken et al. (2009b)
Barley	USA	*Diuraphis noxia*	aphid population growth	2009	"We would like to emphasize that it is not the objective of this study to build predictive models for RWA population dynamics; instead, we try to explore the intrinsic properties of RWA population growth by building a catastrophe theory model with the population intrinsic rate ofgrowth (rm) various environmental conditions."	MA and Bechinski (2009)
Barley	Czech Republic	*Rhopalosiphum padi*	Barley yellow dwarf virus (BYDW) incidence	2019	"Here, our aim was to explain aphid occurrences using Czech weather data and to develop predictive models that could enable farmers in Central Europe to predict the aphid migration time, duration, and total aphid numbers in the weeks ahead of their arrival."	Jarošová et al. (2019)
Corn	Egypt	*Rhopalosiphum maidis*	yield loss	2004	"The aim of the present study is to assess the corn yield losses and to build forecasting models of yield related with aphid infestation."	Al-Eryan and El-Tabbakh (2004)
Corn, sorghum, wheat, wheat volunteer	France	*Rhopalosiphum padi*	aphid population dynamics	2021	"In this study, we used a spatially explicit simulation model to (1) determine how climate, pest immigration, and spatial–temporal variation in habitat availability influence seasonal variation of pest populations in both individual crops and at the landscape level, and (2) explore agronomic scenarios to identify potential effects of changes in practices on aphid populations and help inform future decisionmaking."	Thierry et al. (2021)
Sorghum	USA	*Melanaphis sacchari*	spatio-temporal infestation dynamics / invasion	2020	"We describe a prototype of a computational framework that could be used to forecast sugarcane aphid invasions of sorghum in near-real-time, as supported by timely field reports on current aphid infestation status."	Koralewski et al. (2020a)
Sorghum	USA	*Melanaphis sacchari*	spatio-temporal infestation dynamics / invasion	2020	"We use an integrated ecological model to simulate local and regional infestation dynamics of sugarcane aphids, *Melanaphis sacchari* (Zehntner) (Hemiptera: Aphididae), on sorghum, *Sorghum bicolor* (L.) Moench (family Poaceae), in the southern to central Great Plains of the United States. Local dynamics of aphid populations on sorghum are simulated by a spatially explicit, individual-based model, whereas regional aphid migration is simulated by an atmospheric model that computes inert air particle (aphid) transport, dispersion, and deposition."	Koralewski et al. (2020b)
Wheat	UK	*Metopolophium dirhodum*	peak population density	1990	"In this paper we use winter and spring temperatures to predict the time when *M. dirhodum* populations on wheat will stop increasing the following summer."	Howard and Dixon (1990)
Wheat	China	*Sitobion avenae, Schizaphis graminum, Rhopalosiphum padi*	aphid population dynamics	2014	"In this study, we developed a butterfly catastrophe model for the aphid population as a function of four controlling factors: natural enemy, weather factor, pesticide effect and carrying capacity (crop condition)."	Wu et al. (2014)
Wheat	Denmark	Cereal aphids	spatial aphid population growth	2020	"In this study, we have sampled aphid populations in a spatial set-up during both the initial and the epidemic phase to fit a population growth model. More specifically, we have developed a spatio-temporal stochastic aphid population growth model and fitted the model to empirical spatial time series aphid population data using a Bayesian hierarchical fitting procedure."	Damgaard et al. (2020)

Crop	Country	Aphid species	Aim / scope	Year	Stated purpose	Reference
**Cereals (continued)**						
Winter barley	UK	*Rhopalosiphum padi*	aphid population dynamics	2000	"The aims of this paper are to utilize simulation analysis techniques to develop a model describing the population development of *R. padi* , to use the model to examine the relative importance ofvarious population processes in pest dynamics and to determine whether the model could be used as a basis for forecasting aphid outbreaks."	Morgan (2000)
Winter cereals	England	*Rhopalosiphum spp., Sitobion spp.*	Barley yellow dwarf virus (BYDV) incidence	1992	"A computer model is described which simulates the spread of barley yellow dwarf virus (BYDV) by aphids in winter cereals in SW England."	Kendall et al. (1992)
Winter wheat	EU	*Sitobion avenae*	aphid population dynamics	1982	"Out aim in this monograph are to to explain the population development if *S. avenae* on cereals and to indicate how cereal aphid outbreaks might be predicted."	Carter et al. (1982)
Winter wheat	UK	*Sitobion avenae*	aphid population dynamics	1985	"The simulation model described below was developed to gain understanding of the popula- tion dynamics of the grain aphid, *Sitobion avenae* (F.), in order to develop a reliable forecasting scheme."	Carter (1985)
Winter wheat	UK, Netherlands	*Sitobion avenae*	peak population density	1986	"This paper sets out to test the hypothesis that both the population density of *S. avenae* on wheat and its observed rate of increase are important predictors of peak population size. It also aims to test whether they can be used together to forecast peak *S. avenae* density, and whether the anomalous suction trap forecasts cited above can be accounted for."	Entwistle and Dixon (1986)
Winter wheat	Netherlands	*Sitobion avenae*	aphid damage / yield loss	1986	"Therefore, to quantify the effect of various dynamic reduction processes on growth and development of winter wheat, a simulation approach was adopted, using quantitative data from laboratory studies."	van Roermund et al. (1986)
Winter wheat	France	*Sitobion avenae*	aphid abundance	1987	"[…] prevision of *S. avenae* maximal populations on wheat […]"	Dedryver et al. (1987)
Winter wheat	UK, Netherlands	*Sitobion avenae*	yield loss	1987	"A damage model was used to take into account the effect on winter wheat yield of the size, duration and timing of *Sitobion avenae* infestation in summer, and the influence of insecticide applications."	Entwistle and Dixon (1987)
Winter wheat	UK	*Sitobion avenae, Metopolophium dirhodum*	aphid population dynamics, yield loss	1987	"The package gives advice on the control of the aphids Sitobion avenae and *Metopolophium dirhodum.* It is designed to prevent the overuse of chemical spray applications by forecasting aphid populations and then calculating the economics of control."	Mann and Wratten (1991)
Winter wheat	UK	*Metopolophium dirhodum*	aphid population dynamics	1989	"A simulation model was developed, based on information from the literature, to study its population dynamics and damage potential."	Zhou et al. (1989)
Winter wheat	Netherlands	*Sitobion avenae*	aphid damage / yield loss	1991	"The model is used to assess the contribution of various injury components of S. avenae to damage and to evaluate the consequences of the lack of detailed information on some processes in the aphid - winter wheat system for predicted yield."	Rossing (1991a)
Winter wheat	Netherlands	*Sitobion avenae*	aphid damage / yield loss	1991	"Regression models are constructed that relate simulated aphid damage both during various periods of crop development and averaged over the entire post-anthesis phase, to the simulated attainable yield level."	Rossing (1991b)
Winter wheat	Germany	*Sitobion avenae*	summer population size	1994	"The implemented model 'LAUS' for the grain aphid in early summer is presented with its weather-driven run through submodels for wheat microclimate and phenology and for the population dynamics of aphid antagonists."	Friesland (1994)
Winter wheat	Germany	*Sitobion avenae, Rhopalosiphum padi, Metopolophium dirhodum*	aphid population dynamics	1996	"In this report, GTLAUS (version 3.7), a discrete simulation model of wheat -cereal aphid -predator interaction, which can be used for complex ecological studies in a representative tritrophic system of arable farming, is described and presented with selected scenario runs."	Freier et al. (1996)
Winter wheat	UK	*Sitobion avenae*	aphid population dynamics	1997	"This paper describes the derivation of a mechanistic computer model of the interaction between the cereal aphid *Sitobion avenae* and the coccinellid *Coccinella septempunctata,* and its validation using all the limited data available."	Skirvin et al. (1997)

Crop	Country	Aphid species	Aim / scope	Year	Stated purpose	Reference
**Cereals (continued)**						
Winter wheat	USA	*Diuraphis noxia*	aphid damage / yield loss	2006	"Keeping this in view, the present study was undertaken to simulate the effect of Russian wheat aphid and two winter annual grass weeds on winter wheat yield and to demonstrate that economic injury levels can be determined through simulation models."	Chander et al. (2006)
Winter wheat	France	*Sitobion avenae*	aphid population dynamics	2014	"We expose here a detailed spatially explicit model ofaphid population dynamics at the scale of a whole country (Metropolitan France). It is based on convection–diffusionreaction equations, driven by abiotic and biotic factors. The target species is the grain aphid, *Sitobion avenae* F., considering both its winged and apterous morphs."	Ciss et al. (2014)
Winter wheat	China	*Sitobion avenae*	aphid abundance	2019	"Using *Sitobion avenae* and climatological data collected in China, we made use of differential evolution (DE) algorithms to construct FCM models that directly illustrate the effect of climate on wheat aphid abundance."	Zhang et al. (2019)
Winter wheat	China	*Sitobion avenae, Schizaphis graminum, Rhopalosiphum padi*	aphid population dynamics	2020	"In this paper, we built a generalized population dynamics (GPD) model with three external factors (meteorological conditions, natural enemies and insecticide) to provide a more reasonable explanation for the choice of catastrophe models"	Li et al. (2020a)
Winter wheat, s	Denmark	*Sitobion avenae, Rhopalosiphum padi*	spring migration	2006	"The present study aimed to describe two separate models based on temperature that predict the spring migration of the grain aphid to winter wheat crops and the bird cherry-oat aphid to spring barley crops."	Hansen (2006)
Winter wheat, winter barle)	Germany	*Sitobion avenae, Rhopalosiphum padi*	Validation of GETLAUS02; SIMLAUS; LAUS	2009	"In this paper, three models for describing the population dynamics of cereal aphids were validated, using data collected during 1993 to 2006 from different locations in Germany. The aim of this study was to compare the performance of the three models SIMLAUS [...], LAUS [...] and GETLAUS01 [...], with respect to their usefulness as decision support systems in integrated pest management."	Klueken et al. (2009a)
**Cotton**						
Cotton	USA	*Aphis gossypii*	aphid population dynamics	2008	"Thus, the overall objective of the present study was to test the significance of the main and interaction effects of different levels of nitrogen fertilizer and irrigation water on the population dynamics of the cotton aphid."	T. I. Matis et al. (2008)
Cotton	India	*Aphis gossypii*	aphid population growth / pest risk	2019	"The fundamental objective of this study was to develop a comprehensive temperature-based population model for *A. gossypii* that predicts pest's population growth potential under variable temperature conditions and to map the risk for its establishment and survival in various geographical areas of India using GIS-aided tools."	Nagrare et al. (2021)
Cotton	China	*Aphis gossypii*	aphid population dynamics	2018	"The study aimed at improving quantitative insight in the dynamic behaviour and sensitivities of the aphid−ladybeetle population system by developing a comprehensive simulation model."	Xia et al. (2018)
**Herbs**						
Fennel	Brazil	*Hyadaphis foeniculi*	duration of aphid development	2015	"Assessing the relevance of the most realistic phenological models that make it possible to predict outbreaks of *H. foeniculi* so that they can be controlled in regions potentially suitable for growing fennel, this study was conducted with the aim of developing current knowledge of the thermal characteristics of *H. foeniculi* populations collected in the region of Brejo da Paraíba, Brazil, as a prerequisite for developing an integrated pest management programme."	Malaquias et al. (2015)

Crop	Country	Aphid species	Aim / scope	Year	Stated purpose	Reference
**Hop**						
Hop	UK	*Phorodon humili*	spring migration	1983	"The dates of the beginning and end of the spring migration of *P. humuli* […] were linearly regressed on temperature, rainfall and sunshine for varying periods."	Thomas et al. (1983)
Hop	UK	*Phorodon humili*	spring migration	1995	"This study investigated the relationship between the flowering phenology of two host plum species [Myrobalan *Prunus cerasifera* Ehrh. and Victoria *Prunus domestic* (L.)], on which *P. humuli* overwinters (recorded at East Malling and Brogdale, Kent), mean temperature, and the start of migration of *P. humuli* to hops in spring, as measured by suction traps [...]."	Worner et al. (1995)
**Legumes**						
Legumes	Australia	*Aphis craccivora*	aphid population dynamics, Subterranean Clover Stunt Virus (SCSV) incidence	1974	"A simulation model describing the interactions of various components of the biology of cowpea aphid *(Aphis craccivora* Koch), its host plant, Subterranean Clover Stunt Virus (SCSV) and climate is presented in this paper."	Gutierrez et al. (1974)
Legumes	USA	*Acyrthosiphon pisum*	individual aphid development	1984	"The pea aphid models [2,3] above describe the growth and development of individual aphids under varying conditions of food and temperature, as well as the effects of food and temperature on age-specific life-table statistics."	Gutierrez and Baumgaertner (1984)
Alfalfa	USA	*Acyrthosiphon pisum, Acyrthosiphon kondoi*	aphid population dynamics	1984	"The field population dynamics of pea aphid (*Acyrthosiphon pisum*) and blue alfalfa aphid (*A. kondoi*) in alfalfa (*Medicago sativa*), as influenced by weather, competitors (Egyptian alfalfa weevil = EAW, *Hypera brunneipennis*), predation from coccinellids (*Hippodamia convergens*) and harvesting practices, are examined with a stochastic multitrophic level simulation model."	Gutierrez et al. (1984)
Beans	Germany	*Aphis fabae, Myzus persicae, Acyrtosiphum pisum*	summer population size / general abundance	1997	"This study investigates whether (a), this was a true endogenous pattern, (b), what other factors influence the aphid's densities, and (c), whether there is a simple method of forecasting the abundance of all three species of aphid at Rostock, Germany."	Thacker et al. (1997)
Broad beans	UK	*Aphis fabae*	aphid infestation	1981	"In the present paper, we use these crop samples to compare the accuracy of forecasts based on egg counts and spring populations of *A. fabae* on *E. europaeus* with those based on the aerial monitoring samples. Secondly. we relate the several sequential samples - the autumn migration. the over-wintering egg counts. the spring populations on E. europaeus (fundatrigeniae) and the spring migration, to early infestations in field bean crops."	Way et al. (1981)
Broad beans	UK	*Aphis fabae*	aphid infestation	1994	"This paper describes the decision problem of controlling *A. fabae* , the knowledge acquisition and engineering, and the encoding of the information into a computer program."	Knight and Cammell (1994)
Luoin	Australia	Colonizers: *Aphis craccivora, Acyrthosiphon kondoi, Myzus persicae* ; Non-colonizers: Lipaphis erysimi, Rhopalosiphum maidis Rhopalosiphum padi	aphid immigration into lupin crops, Cucumber mosaic virus (CMV) incidence	2004	"A simulation model was developed to forecast aphid outbreaks and epidemics of CMV in lupin crops […]."	Thackray et al. (2004)
Lupin	Australia	Colonizers: *Aphis craccivora, Acyrthosiphon kondoi, Myzus persicae; Non-colonizers: Lipaphis erysimi, Rhopalosiphum maidis Rhopalosiphum padi*	aphid immigration into lupin crops, Bean yellow mosaic virus (BYMV) incidence	2008	"A hybrid mechanistic/statistical model was developed to predict vector activity and epidemics of vector-borne viruses spreading from external virus sources to an adjacent crop."	Maling et al. (2008)

Crop	Country	Aphid species	Aim / scope	Year	Stated purpose	Reference
			**Legumes (continued)**			
Peas	UK	*Acyrthosiphon pisum*	aphid population dynamics, initial flight activity	1999	"The objectives of this work were to establish why both the time ofdevelopment and the abundance ofpea aphid populations on pea crops varies. A forecast of the time of first appearance would allow preparation for spraying in advance of the time at which the aphids appeared on the crop so that insecticide could be applied promptly, whilst understanding the reasons for the variability of infestation would mean that the effect of different aspects of husbandry, such as time of sowing, could be assessed."	McVean et al. (1999)
Peas	Australia	*Acyrthosiphon kondoi, Lipaphis erysimi, Myzus persicae, Rhopalosiphum padi*	Pea seed-borne mosaic virus (PSbMV) incidence	2017	"The aim of this study was to develop a model that predicts aphid numbers, PSbMV crop incidence and, ultimately, yield and economic loss, to inform end-user decisions about the need to source seed with minimal infection, which cultivar to use and the utilization of cultural control measures."	Congdon et al. (2017)
Soybean	USA	*Aphis glycines*	aphid population dynamics	2005	"In this article, we investigate the population growth of this *A. glycines* in soybean fields in Illinois at the township level."	Onstad et al. (2005)

Crop	Country	Aphid species	Aim / scope	Year	Stated purpose	Reference
			**Potato**			
Potato	USA	*Myzus persicae*	aphid population dynamics	1979	"The objectives were to develop a model which was compatible with the microcomputer delivery system and sufficiently accurate to forecast [*Myzus persicae*] populations as they reached economically damaging levels."	Whalon and Smilowitz (1979a)
Potato	Canada	*Myzus persicae*	aphid population dynamics	1979	"A temperature-dependent model for predicting green peach aphid population development in potato fields was devised and tested."	Whalon and Smilowitz (1979b)
Potato, cereals	Scotland	*Myzus persicae, Macrosiphum euphorbiae, Aulacorthum solani, Sitobion avenae, Sitobion fragariae, Metopolophium dirhodum, Metapolophium festucae, Rhopalosiphum padi, Rhopalosiphum insertum*	initial flight activity	1980	"The establishment of the 12.2 m suction traps by the Rothamsted Insect Survey offers a new approach to forecasting the overwintering success of certain aphid species based on the first date, in any year, when a species is first caught in a particular trap."	Turl (1980)
Potato	Scotland	*Myzus persicae*	immigration into potato crops	1987	"The results of a six year field study of *Myzus persicae* overwintering are reported wich show that the success of anholocyclic overwintering is a function of winter temperature. […] This relationship can be used to forecast the size of the immigration into seed potato crops (one summer hoest) early enough to allow the application of control measures."	Walters (1987)
Potato	Sweden	*Acyrthosiphon pisum, Aphis fabae gr., Aphis nasturtii, Aphis frangulae, Brevicoryne brassicae, Metopolophium dirhodum, Myzus persicae, Rhopalosiphum padi, Sitobion avenae. Others: Rhopalosiphum padi, Brachycaudus helichrysi, Acyrthosiphon pisum, Phorodon humuli*	Potato virus Y (PVY) incidence	1992	"A dynamic simulation model for PVY has been designed for predicting the incidence of PVY."	Sigvald (1992)

Crop	Country	Aphid species	Aim / scope	Year	Stated purpose	Reference
**Potato (continued)**						
Potato	Peru	Potato aphids	Potato virus incidence	1995	"A model (EPIVIT) was developed for the simulation of potato harvest infection (% infected tubers) with a contct- or aphid-transmitted virus. [...] The purpose of the model construction was to otain a tool for forecasting epidemics of the most important viruses of the potato crop in the Andes."	Bertschinger et al. (1995)
Potato	USA	*Myzus persicae*	aphid population dynamics	1999	" […] the model describes the interaction among the green peach aphid population, its predators, and abiotic factors (e.g. temperature). The model includes functions for population growth of the aphid and its predator complex."	Ro and Long (1999)
Potato	Hungary	*Myzus persicae, Macrosiphum euphorbiae, Acyrthosiphum pisum, Aphis nasturtii, Aphis fabae, Phorodon humuli, Rhopalosiphum padi, Metopolophium dirhodum, Brachycaudus helichrysi, Brachycaudus cardui*	Potato virus (PYV, PLRV) incidence	2002	"This study presents relationships based on long-term aphid flight activity, flight activity of PVY vector aphids, cumulative vector intensity and the rate of PVY and PLRV infection of progeny tubers of different cultivars in Hungary."	Basky (2002)
Potato	Swiss	*Myzus persicae, Brachycaudus helichrysi, Aphis fabae, Aphis spp., Phorodonhumuli, Rhopalosiphum padi, Acyrthosiphon pisum*	Potato virus Y (PVY) incidence	2015	"The ultimate aim of this study was to develop a decision-support system capable of forecasting virus spread during the current season using trap data of aphid flights."	Steinger et al. (2015)
**Sugarbeet**						
Sugar beet	UK	*Myzus persicae*	Sugar beet Virus yellows (BVY, BMYV, BChV) incidence	1965	"The results of the following investigation reflect the effect of the various weather elements on the aphid but population changes have been ignored (reliable systematic counts are relatively recent) and only their effect on the spread of the yellows virus is considered."	Hurst (1965)
Sugar beet	USA	*Myzus persicae*	aphid population dynamics	1978	"A model to evaluate potential reductive impact of populations of green peach aphid, *Myzus persicae* (Sulzer), by predators was generalized to include effects of temperature on the rate of aphid reproduction and the functional response of the predator complex."	Tamaki and Long (1978)
Sugar beet	UK	*Myzus persicae*	Sugar beet Virus yellows (BVY, BMYV, BChV) incidence	1989	"The forecast uses multiple regression techniques and includes information on the size of the pool of virus sources, the weather that limits the numbers of aphid vectors and the activity of these vectors in spring."	Harrington et al. (1989)
Sugar beet	UK	*Myzus persicae*	Sugar beet virus yellows (BMYV, BYV) incidence	1998	"The objective of this investigation was to develop a model as the basis of a new virus yellows forecasting system."	Werker et al. (1998)
Sugar beet	Netherlands	*Myzus persicae*	Sugar beet Virus yellows (BtMV) secondary spread	2000	"The studies described were aimed to answer the questions (1) how does the inoculation date affect the spread of BtMV in sugar beet; (2) in which way is the size and pattern of the spread related to abundance, time profile and species spectrum of vectors; and (3) can the spread be described by a simple mechanistic simulation model, using accepted principles of disease epidemics."	Dusi et al. (2000)
Sugar beet	UK	*Myzus persicae*	Sugar beet Virus yellows (BVY, BMYV, BChV) incidence	2004	"This paper reviews and examines the procedures in the decisionmaking processes and the likely future developments in controlling *M. persicae* and, consequently, virus yellows in sugar beet crops in relation to evolving control methods and mathematical modelling."	Qi et al. (2004)

Crop	Country	Aphid species	Aim / scope	Year	Stated purpose	Reference
**Others**						
Lime tree	UK	*Eucallipterus tiliae*	aphid population dynamics	1979	"The results from a long-term field study (10 years) and laboratory experiments have been used to develop a simulation model of lime aphid populations."	Dixon and Barlow (1979)
Lime tree	New Zealand, UK	*Eucallipterus tiliae*	aphid population dynamics	1980	"This further book in a series of monographs on computer simulation in agriculture and related sciences [...] describes a systems approach to the population dynamics of *Eucallipterus tiliae* (L.), an aphid specific to most members of the genus *Tilia* ."	Barlow and Dixon (1980)
Lime tree	New Zealand, UK	*Eucallipterus tiliae*	aphid population dynamics	1981	"This paper considers two approaches to the modelling of aphid populations. Using the lime aphid (*Eucallipterus tiliae* L.) as an example, it discusses the well-established role of detailed simulation models in the study of aphid population dynamics then considers the possible application of a simple, general herbivore/plant model to aphids."	Barlow (1981)
Orchards (apple)	Switzerland	*Dysaphis plantaginea*	time of egg hatching	2006	"The objective of the present study was to develop a basis for a reliable, temperature-driven tool to forecast the phenology of *D. plantaginea* in order to facilitate decision making of apple growers with respect to aphid control before bloom."	Graf et al. (2006)
Orchards (pecan)	India	*Lipaphis erysimi*	aphid population dynamics	2007	"This paper develops a deterministic and a stochastic population size model based on power-law kinetics for the black-margined pecan aphid."	Matis et al. (2007)
Pepper	Canada	-	virus disease incidence	1978	"The objectives of this study were to identify the meteorological variables during 1970-1977 that relate to virus disease incidence in this crop in the Niagara Peninsula and ultimateley to forecast virus disease incidence or at least to predict, in advance, years of high virus risk based primarily on weather parameters.	Kemp and Troup (1978)
Sitka spruce	UK	*Elatobium abietinum*	aphid population dynamics	2010	"Our aim was to find the best model to explain year-to year variation in *E. abietinum* peak abundance."	Day et al. (2010)
Sitka spruce	Canada	*Elatobium abietinum*	aphid population dynamics	2022	"Our objective here is to construct a transparent mechanistic model ofthe green spruce aphid and interface this with a long-established mechanistic forest ecosystem simulation model, the Edinburgh Forest Model [...]"	Thornley and Newman (2022)
-	Italy	Various (differenciated by holocyclic, anholocyclic and holo-anholocyclic species)	spring population size	2001	"The primary objective of this study was to quantify the effects of autumn and winter meteorological variables on the aphid species populations the following spring."	Rongai et al. (2001)
-	UK	Various	initial flight activity	1991	"Studies have related suction trapping data to weather data […]. Results presented in this paper extend these studies to more sites, species and years and use more weather variables in an improved multiple regression approach."	Harrington et al. (1991)
-	UK	*Myzus persicae*	initial flight activity	2009	"This paper describes the multiple regression technique in greater detail and assesses the abilities of the models using this technique, and using simple regression with mean temperature, to predict the date of the first record of *Myzus persicae* in the Rothamsted suction trap in the years from 1989 to 1992."	Howling et al. (1993)

References

Al-Eryan, M. A. S., and El-Tabbakh, S. S. (2004). Forecasting yield of corn, *Zea mays* infested with corn leaf aphid, *Rhopalosiphum maidis* . *J. Appl. Entomol.* 128, 312–315. doi: 10.1111/j.1439-0418.2004.00852.x

Barlow, N. D. (1981). Modelling aphid populations. *N. Z. J. Ecol.* 4, 52–55.

Barlow, N. D., and Dixon, A. F. G. (1980). *Simulation of lime aphid population dynamics* . Wageningen: Centre for Agricultural Publ. and Documentation.

Barton, M., Parry, H., Ward, S., Hoffmann, A. A., Umina, P. A., van Helden, M., et al. (2021). Forecasting impacts of biological control under future climates: mechanistic modelling of an aphid pest and a parasitic wasp. *Ecol. Modell.* 457, 109679. doi: 10.1016/j.ecolmodel.2021.109679

Basky, Z. (2002). The relationship between aphid dynamics and two prominent potato viruses (PVY and PLRV) in seed potatoes in Hungary. *Crop Prot.* 21, 823–827. doi: 10.1016/S0261-2194(02)00045-5

Bertschinger, L., Keller, E. R., and Gessler, C. (1995). Development of EPIVIT, a simulation model for contact- and aphid-transmitted potato viruses. *Phytopathology* 85, 801. doi: 10.1094/Phyto-85-801

Carter, N. (1985). Simulation modelling of the population dynamics of cereal aphids. *BioSystems* 18, 111–119. doi: 10.1016/0303-2647(85)90064-4

References (continued)

Carter, N., Dixon, A. F. G., and Rabbinge, R. (1982). *Cereal aphid populations: biology, simulation and prediction*. Wageningen: Centre for Agricultural Publ. and Doc.

Carter, N., and Rabbinge, R. (1980). Simulation models of the population development of Sitobion avenae. *IOBC/WPRS Bull.* 3, 93–98.

Chander, S., Ahuja, L. R., Peairs, F. B., Aggarwal, P. K., and Kalra, N. (2006). Modeling the effect of Russian wheat aphid, *Diuraphis noxia* (Mordvilko) and weeds in winter wheat as guide to management. *Agric. Syst.* 88, 494–513. doi: 10.1016/j.agsy.2005.07.009

Chattopadhyay, C., Agrawal, R., Kumar, A., Singh, Y. P., Roy, S. K., Khan, S. A., et al. (2005). Forecasting of *Lipaphis erysimi* on oilseed Brassicas in India—a case study. *Crop Prot.* 24, 1042–1053. doi: 10.1016/j.cropro.2005.02.010

Ciss, M., Parisey, N., Moreau, F., Dedryver, C.-A., and Pierre, J.-S. (2014). A spatiotemporal model for predicting grain aphid population dynamics and optimizing insecticide sprays at the scale of continental France. *Environ Sci Pollut Res* 21, 4819–4827. doi: 10.1007/s11356-013-2245-1

Congdon, B. S., Coutts, B. A., Jones, R. A. C., and Renton, M. (2017). Forecasting model for *Pea seed-borne mosaic virus* epidemics in field pea crops in a Mediterranean-type environment. *Virus Res.* 241, 163–171. doi: 10.1016/j.virusres.2017.05.018

Damgaard, C., Bruus, M., and Axelsen, J. A. (2020). The effect of spatial variation for predicting aphid outbreaks. *J. Appl. Entomol.* 144, 263–269. doi: 10.1111/jen.12724

Day, K. R., Ayres, M. P., Harrington, R., and Kidd, N. A. C. (2010). Interannual dynamics of aerial and arboreal green spruce aphid populations. *Popul. Ecol.* 52, 317–327. doi: 10.1007/s10144-009-0190-0

Dedryver, C. A., Fougeroux, A., La Messeliere, C. de, and Pierre, J. S. (1987). Resultats Preliminaries Concernant L'etablissement D'un Modele De Previsions des risques de pullulation de *Sitobion aenae* F. sur ble au printemps dans le bassin Parisien et le nord de la France. *IOBC/WPRS Bull.* 10, 133–142.

Dixon, A. F. G., and Barlow, N. D. (1979). Population regulation in the lime aphid. *Zool. J. Linn. Soc.* 67, 225–237. doi: 10.1111/j.1096-3642.1979.tb01113.x

Duffy, C., Fealy, R., and Fealy, R. M. (2017). An improved simulation model to describe the temperature-dependent population dynamics of the grain aphid, *Sitobion avenae*. *Ecol. Modell.* 354, 140–171. doi: 10.1016/j.ecolmodel.2017.03.011

Dusi, A. N., Peters, D., and van der Werf, W. (2000). Measuring and modelling the effects of inoculation date and aphid flights on the secondary spread of Beet mosaic virus in sugar beet. *Ann. Appl. Biol.* 136, 131–146. doi: 10.1111/j.1744-7348.2000.tb00018.x

Entwistle, J. C., and Dixon, A. F. G. (1986). Short-term forecasting of peak population density of the grain aphid (*Sitobion avenae*) on wheat. *Ann. Appl. Biol.* 109, 215–222. doi: 10.1111/j.1744-7348.1986.tb05313.x

Entwistle, J. C., and Dixon, A. F. G. (1987). Short-term forecasting of wheat yield loss caused by the grain aphid (*Sitobion avenae*) in summer. *Ann. Appl. Biol.* 111, 489–508. doi: 10.1111/j.1744-7348.1987.tb02007.x

Fabre, F., Dedryver, C. A., Leterrier, J. L., and Plantegenest, M. (2003). Aphid abundance on cereals in autumn predicts yield losses caused by *Barley yellow dwarf virus*. *Phytopathology* 93, 1217–1222. doi: 10.1094/PHYTO.2003.93.10.1217

Fabre, F., Pierre, J. S., Dedryver, C. A., and Plantegenest, M. (2006). *Barley yellow dwarf disease* risk assessment based on Bayesian modelling of aphid population dynamics. *Ecol. Modell.* 193, 457–466. doi: 10.1016/j.ecolmodel.2005.08.021

Freier, B., Triltsch, H., and Roßberg, D. (1996). GTLAUS - Ein Modell für die Interaktion Weizen - Getreideblattläuse - Prädatoren und dessen Nutzung für komplexe agrarökologische Studien. *J. Plant Dis. Prot.* 103, 543–554.

Friesland, H. (1994). 'LAUS', ein Prognosemodell für den Getreideblattlausbefall als Beispiel aus dem agrarmeteorologischen Softwarepaket 'AMBER'. *Nachrichtenbl. Deut. Pflanzenschutzd.* 46, 287–291.

Gosselke, U., Triltsch, H., Roßberg, D., and Freier, B. (2001). GETLAUS01—the latest version of a model for simulating aphid population dynamics in dependence on antagonists in wheat. *Ecol. Modell.* 145, 143–157. doi: 10.1016/S0304-3800(01)00386-6

Graf, B., Hopli, H. U., Hohn, H., and Samietz, J. (2006). Temperature effects on egg development of the rosy apple aphid and forecasting of egg hatch. *Entomol. Exp. Appl.* 119, 207–211. doi: 10.1111/j.1570-7458.2006.00411.x

Gutierrez, A. P., and Baumgaertner, J. U. (1984). Multitrophic models of predator-prey energetics: II. A realistic model of plant-herbivore-parasitoid-predator interactions. *Can. Entomol.* 116, 933–949. doi: 10.4039/Ent116933-7

Gutierrez, A. P., Baumgaertner, J. U., and Summers, C. G. (1984). Multitrophic models of predator-prey energetics: III. A case study in an alfalfa ecosystem. *Can. Entomol.* 116, 950–963. doi: 10.4039/Ent116950-7

Gutierrez, A. P., Nix, H. A., Havenstein, D. E., and Moore, P. A. (1974). The Ecology of Aphis craccivora Koch and Subterranean Clover Stunt Virus in South-East Australia. III. A regional perspective of the phenology and migration of the cowpea aphid. *J Appl Ecology* 11, 21–35. doi: 10.2307/2402002

Hansen, L. M. (2006). Models for spring migration of two aphid species *Sitobion avenae* (F.) and *Rhopalosiphum padi* (L.) infesting cereals in areas where they are entirely holocyclic. *Agric. Forest Ent.* 8, 83–88. doi: 10.1111/j.1461-9563.2006.00289.x

Harrington, R., Dewar, A. M., and George, B. (1989). Forecasting the incidence of virus yellows in sugar beet in England. *Ann. Appl. Biol.* 114, 459–469. doi: 10.1111/j.1744-7348.1989.tb03361.x

Harrington, R., Howling, G. G., Bale, J. S., and Clark, S. (1991). A new approach to the use of meteorological and suction trap data in predicting aphid problems. *EPPO Bull.* 21, 499–505. doi: 10.1111/j.1365-2338.1991.tb01281.x

Honěk, A., Martinková, Z., Brabec, M., and Saska, P. (2019). Predicting aphid abundance on winter wheat using suction trap catches. *Plant Prot. Sci.* 56, 35–45. doi: 10.17221/53/2019-PPS

Howard, M. T., and Dixon, A. F. G. (1990). Forecasting of peak population density of the rose grain aphid *Metopolophium dirhodum* on wheat. *Ann. Appl. Biol.* 117, 9–19. doi: 10.1111/j.1744-7348.1990.tb04190.x

Howling, G. G., Harrington, R., Clark, S. J., and Bale, J. S. (1993). The use of multiple regression via principal components in forecasting early season aphid (Homoptera: Aphididae) flight. *Bull. Ent. Res.* 83, 377–381. doi: 10.1017/S0007485300029278

Hughes, R. D., and Gilbert, N. (1968). A model of an aphid population-a general statement. *J. Anim. Ecol.* 37, 553. doi: 10.2307/3074

Hurst, G. W. (1965). Forcasting the severity of sugar beet yellows. *Plant. Pathol.* 14, 47–53. doi: 10.1111/j.1365-3059.1965.tb00274.x

Jarošová, J., Żelazny, W. R., and Kundu, J. K. (2019). Patterns and predictions of *Barley yellow dwarf virus v*ector migrations in Central Europe. *Plant Dis.* 103, 2057–2064. doi: 10.1094/PDIS-11-18-1999-RE

Kemp, W. G., and Troup, P. A. (1978). A weather index to forecast potential incidence of aphid-transmitted virus diseases of peppers in the Niagara Peninsula. *Can. J. Plant Sci.* 58, 1025–1028. doi: 10.4141/cjps78-156

Kendall, D. A., Brain, P., and Chinn, N. E. (1992). A simulation model of the epidemiology of barley yellow dwarf virus in winter sown cereals and its application to forecasting. *J. Appl. Ecol.* 29, 414. doi: 10.2307/2404510

Klueken, A. M., Hau, B., Freier, B., Friesland, H., Kleinhenz, B., and Poehling, H.-M. (2009a). Comparison and validation of population models for cereal aphids. *J. Plant Dis. Prot.* 116, 129–140. doi: 10.1007/BF03356299

References (continued)

Klueken, A. M., Hau, B., Ulber, B., and Poehling, H.-M. (2009b). Forecasting migration of cereal aphids (Hemiptera: Aphididae) in autumn and spring. *J. Appl. Entomol.* 133, 328–344. doi: 10.1111/j.1439-0418.2009.01387.x

Knight, J. D., and Cammell, M. E. (1994). A decision support system for forecasting infestations of the black bean aphid, *Aphis fabae* Scop., on spring-sown field beans, *Vicia faba* . *Comput. Electron. Agric.* 10, 269–279. doi: 10.1016/0168-1699(94)90046-9

Koralewski, T. E., Wang, H.-H., Grant, W. E., Brewer, M. J., Elliott, N. C., Westbrook, J. K., et al. (2020a). Integrating models of atmospheric dispersion and crop-pest dynamics: linking detection of local aphid infestations to forecasts of region-wide invasion of cereal crops. *Annals of the Entomological Society of America* 113, 79–87. doi: 10.1093/aesa/saz047

Koralewski, T. E., Wang, H.-H., Grant, W. E., LaForest, J. H., Brewer, M. J., Elliott, N. C., et al. (2020b). Toward near-real-time forecasts of airborne crop pests: aphid invasions of cereal grains in North America. *Comput. Electron. Agric.* 179, 105861. doi: 10.1016/j.compag.2020.105861

Kurppa, S. (1989). Predicting outbreaks of *Rhopalosiphum padi* in Finland. *Ann. Agr. Fenn.* 28, 333–347.

Lankin-Vega, G., Worner, S. P., and Teulon, D. A. J. (2008). An ensemble model for predicting *Rhopalosiphum padi* abundance. *Entomol. Exp. Appl.* 129, 308–315. doi: 10.1111/j.1570-7458.2008.00778.x

Li, Y., Hu, Z., Li, Z., Kong, Y., Piyaratne, M. K. D. K., Wang, B., et al. (2020a). Generalized population dynamics model of aphids in wheat based on catastrophe theory. *BioSystems* 198, 104217. doi: 10.1016/j.biosystems.2020.104217

Li, Y., Li, Z., Zhao, L., Hu, Z., and Zhao, H. (2020b). Development of a wheat aphid population dynamics model based on cusp catastrophe theory. *Int. J. Biomath.* 13, 2050078. doi: 10.1142/S1793524520500783

Ma, Z., and Bechinski, E. J. (2008). A survival-analysis-based simulation model for Russian wheat aphid population dynamics. *Ecol. Modell.* 216, 323–332. doi: 10.1016/j.ecolmodel.2008.04.011

Ma, Z. S., and Bechinski, E. J. (2009). An approach to the nonlinear dynamics of Russian wheat aphid population growth with the cusp catastrophe model. *Entomol. Res.* 39, 175–181. doi: 10.1111/j.1748-5967.2009.00216.x

Malaquias, J. B., Ramalho, F. S., Lira, A. C. S., Oliveira, F. Q., Fernandes, F. S., Zanuncio, J. C., et al. (2015). Estimating the development of the fennel aphid, *Hyadaphis foeniculi* (Passerini) (Hemiptera: Aphiidae), using non-linear models. *Pest Manag. Sci.* 71, 744–751. doi: 10.1002/ps.3845

Maling, T., Diggle, A. J., Thackray, D. J., Siddique, K. H. M., and Jones, R. A. C. (2008). An epidemiological model for externally sourced vector-borne viruses applied to *Bean yellow mosaic virus* in lupin crops in a Mediterranean-type environment. *Phytopathology* 98, 1280–1290. doi: 10.1094/PHYTO-98-12-1280

Maling, T., Diggle, A. J., Thackray, D. J., Siddique, K. H. M., and Jones, R. A. C. (2010). An epidemiological model for externally acquired vector-borne viruses applied to *Beet western yellows virus* in *Brassica napus* crops in a Mediterranean-type environment. *Crop Pasture Sci.* 61, 132. doi: 10.1071/CP09180

Mann, B. P., and Wratten, S. D. (1991). A computer-based advisory system for cereal aphids-field-testing the model. *Ann. Appl. Biol.* 118, 503–512. doi: 10.1111/j.1744-7348.1991.tb05340.x

J. H. Matis, T. R. Kiffe, T. I. Matis, and C. Chattopadhyay (2008). Generalized aphid population growth models with immigration and cumulative-size dependent dynamics. *Math. Biosci.* 215, 137–143. doi: 10.1016/j.mbs.2008.07.007

Matis, J. H., Kiffe, T. R., Matis, T. I., and Stevenson, D. E. (2007). Stochastic modeling of aphid population growth with nonlinear, power-law dynamics. *Math. Biosci.* 208, 469–494. doi: 10.1016/j.mbs.2006.11.004

T. I. Matis, M. N. Parajulee, J. H. Matis, and R. B. Shrestha (2008). A mechanistic model based analysis of cotton aphid population dynamics data. *Agric. Forest Ent.* 10, 355–362. doi: 10.1111/j.1461-9563.2008.00389.x

McVean, R. I. K., Dixon, A. F. G., and Harrington, R. (1999). Causes of regional and yearly variation in pea aphid numbers in eastern England. *J. Appl. Entomol.* 123, 495–502. doi: 10.1046/j.1439-0418.1999.00409.x

Morgan, D. (2000). Population dynamics of the bird cherry-oat aphid, *Rhopalosiphum padi* (L.), during the autumn and winter: a modelling approach. *Agric. Forest Ent.* 2, 297–304. doi: 10.1046/j.1461-9563.2000.00079.x

Nagrare, V. S., Fand, B. B., Naikwadi, B. V., and Deshmukh, V. (2021). Potential risk of establishment and survival of cotton aphid *Aphis gossypii* in India based on simulation of temperature-dependent phenology model. *Int. J. Pest Manag.* 67, 187–202. doi: 10.1080/09670874.2019.1649739

Nematollahi, M. R., Fathipour, Y., Talebi, A. A., Karimzadeh, J., and Zalucki, M. P. (2016). Comparison of degree-day distribution models for predicting emergence of the cabbage aphid on canola. *Crop Prot.* 80, 138–143. doi: 10.1016/j.cropro.2015.11.011

Newman, J. A. (2006). Using the output from global circulation models to predict changes in the distribution and abundance of cereal aphids in Canada: a mechanistic modeling approach. *Glob. Change Biol.* 12, 1634–1642. doi: 10.1111/j.1365-2486.2006.01192.x

Onstad, D. W., Fang, S., and Voegtlin, D. J. (2005). Forecasting seasonal population growth of *Aphis glycines* (Hemiptera: Aphididae) in soybean in Illinois. *J Econ Entomol* 98, 1157–1162. doi: 10.1603/0022-0493-98.4.1157

Parker, W. E. (1997). Forecasting the timing and size of field populations of aphids on potato in England and Wales. *Ann. ANPP* 3, 1087–1094.

Parry, H. R., Evans, A. J., and Morgan, D. (2006). Aphid population response to agricultural landscape change: a spatially explicit, individual-based model. *Ecol. Modell.* 199, 451–463. doi: 10.1016/j.ecolmodel.2006.01.006

Pierre, J. S., and Dedryver, C. A. (1984). Un modele de regression multiple applique a la prevision des pullulations d'un puceron des cereales, *Sitobion avenae* F., sur ble d'hiver. *Acta Oecol.* 5, 153–172.

Piyaratne, M., Zhao, H., and Meng, Q. (2013). APHIDSim: A population dynamics model for wheat aphids based on swallowtail catastrophe theory. *Ecol. Modell.* 253, 9–16. doi: 10.1016/j.ecolmodel.2012.12.032

Qi, A., Dewar, A. M., and Harrington, R. (2004). Decision making in controlling virus yellows of sugar beet in the UK. *Pest Manag. Sci.* 60, 727–732. doi: 10.1002/ps.871

Rao, B. B., Ramaraj, A. P., Chattopadhyay, C., Prasad, Y. G., and Rao, V. U. M. (2012). Predictive model for mustard aphid infestation for eastern plains of Rajasthan. *J. Agrometeorol.* 14, 60–62. doi: 10.54386/jam.v14i1.1385

Rao, B. B., Rao, V. U. M., Nair, L., Prasad, Y. G., Ramaraj, A. P., and Chattopadhyay, C. (2014). Mustard aphid infestation in India: development of forewarning models. *J. Environ. Biol.* 35, 683–688.

Raworth, D. A. (1984). Population dynamics of the cabbage aphid, *Brevicoryne brassicae* (Homoptera: Aphididae) at Vancouver, British Columbia: V. A simulation model. *Can. Entomol.* 116, 895–911. doi: 10.4039/Ent116895-6

Ro, T. H., and Long, G. E. (1999). GPA-Phenodynamics, a simulation model for the population dynamics and phenology of green peach aphid in potato: formulation, validation, and analysis. *Ecol. Modell.* 119, 197–209. doi: 10.1016/S0304-3800(99)00053-8

Rongai, D., Bellocchi, G., and Burzi, P. (2001). Effect of autumn and winter meteorological variables on spring aphid populations in the Po valley, Northern Italy. *J. Appl. Entomol.* 125, 437–441. doi: 10.1046/j.1439-0418.2001.00573.x

Rossing, W. A. H. (1991a). Simulation of damage in winter wheat caused by the grain aphid *Sitobion avenae* . 2. Construction and evaluation of a simulation model. *Eur. J. Plant Pathol.* 97, 25–54. doi: 10.1007/BF01995782

References (continued)

Rossing, W. A. H. (1991b). Simulation of damage in winter wheat caused by the grain aphid *Sitobion avenae*. 3. Calculation of damage at various attainable yield levels. *Eur. J. Plant Pathol.* 97, 87–103. doi: 10.1007/BF01974272

Shonga, E., and Getu, E. (2021). Population dynamics of cabbage aphid, *Brevicoryne brassicae* L. (Homoptera: Aphididae) in relation to weather factors on major brassica crops in central rift valley of Ethiopia: baseline studies for the management of the pest. *Int. J. Trop. Insect Sci.* 41, 455–462. doi: 10.1007/s42690-020-00226-4

Sigvald, R. (1992). Progress in aphid forecasting systems. *Eur. J. Plant Pathol.* 98, 55–62. doi: 10.1007/BF01974472

Skirvin, D. J., Perry, J. N., and Harrington, R. (1997). A model describing the population dynamics of *Sitobion avenae* and *Coccinella septempunctata*. *Ecol. Modell.* 96, 29–39. doi: 10.1016/S0304-3800(96)00048-8

Steinger, T., Goy, G., Gilliand, H., Hebeisen, T., and Derron, J. (2015). Forecasting virus disease in seed potatoes using flight activity data of aphid vectors. *Ann. Appl. Biol.* 166, 410–419. doi: 10.1111/aab.12190

Tamaki, G., and Long, G. E. (1978). Predator complex of the Green peach aphid on sugarbeets: expansion of the predator power and efficacy model. *Environ. Entomol.* 7, 835–842. doi: 10.1093/ee/7.6.835

Teulon, D., Stufkens, M., Nicol, D., and Harcourt, S. J. (1999). Forecasting barley yellow dwarf virus in autumn-sown cereals in 1998. *Proc. N.Z. Plant Prot. Conf.* 52, 187–191. doi: 10.30843/NZPP.1999.52.11563

Thacker, J. I., Thieme, T., and Dixon, A. F. G. (1997). Forecasting of periodic fluctuations in annual abundance of the bean aphid: the role of density dependence and weather. *J. Appl. Entomol.* 121, 137–145. doi: 10.1111/j.1439-0418.1997.tb01383.x

Thackray, D. J., Diggle, A. J., Berlandier, F. A., and Jones, R. A. C. (2004). Forecasting aphid outbreaks and epidemics of Cucumber mosaic virus in lupin crops in a Mediterranean-type environment. *Virus Res.* 100, 67–82. doi: 10.1016/j.virusres.2003.12.015

Thackray, D. J., Diggle, A. J., and Jones, R. A. C. (2009). BYDV PREDICTOR: a simulation model to predict aphid arrival, epidemics of Barley yellow dwarf virus and yield losses in wheat crops in a Mediterranean-type environment. *Plant. Pathol.* 58, 186–202. doi: 10.1111/j.1365-3059.2008.01950.x

Tharranum, A. M., Singh, K. K., Pandey, A. C., Singh, Y. P., and Kandpal, B. K. (2020). Evaluation of forewarning models for mustard aphids in different agro-climatic zones of India. *Int. J. Biometeorol.* 64, 445–460. doi: 10.1007/s00484-019-01831-w

Tharranum, M. A., Singh, Y. P., Kandpal, B. K., Singh, K. K., and Pandy, A. C. (2017). Forewarning model development for mustard aphid (*Lipaphis erysimi* Kalt.) at Bharatpur and Hisar. *J. Agrometeorol.* 19, 334–341. doi: 10.54386/jam.v19i4.602

Thierry, H., Monteil, C., Parry, H., and Vialatte, A. (2021). Simulating seasonal drivers of aphid dynamics to explore agronomic scenarios. *Ecosphere* 12. doi: 10.1002/ecs2.3533

Thomas, G. G., Goldwin, G. K., and Tatchell, G. M. (1983). Associations between weather factors and the spring migration of the damson-hop aphid, *Phorodon humuli*. *Ann. Appl. Biol.* 102, 7–17. doi: 10.1111/j.1744-7348.1983.tb02661.x

Thornley, J. H. M., and Newman, J. A. (2022). Climate sensitivity of the complex dynamics of the green spruce aphid-spruce plantation interactions: insight from a new mechanistic model. *PLOS ONE* 17, e0252911. doi: 10.1371/journal.pone.0252911

Turl, L. A. D. (1980). An approach to forecasting the incidence of potato and cereal aphids in Scotland. *EPPO Bull.* 10, 135–141. doi: 10.1111/j.1365-2338.1980.tb02635.x

van Roermund, H., Groot, J., Rossing, W., and Rabbinge, R. (1986). Calculation of aphid damage in winter wheat, using a simulation model. *Mededelingen - Universiteit Gent, Faculteit Landbouwkundige en Toegepaste Biologische Wetenschappen* 51, 1125–1130.

Walters, K. F. A. (1987). *Forecasting the immigration of aphids into potato crops*. Paris.

Walters, K. F. A., and Dewar, A. M. (1986). Overwintering strategy and the timing of the spring migration of the cereal aphids *Sitobion avenae* and *Sitobion fragariae*. *J Appl Ecology* 23, 905. doi: 10.2307/2403943

Way, M. J., Cammell, M. E., Taylor, L. R., and Woiwod, I. P. (1981). The use of egg counts and suction trap samples to forecast the infestation of spring-sown field beans, *Vicia faba*, by the black bean aphid, *Aphis fabae*. *Ann. Appl. Biol.* 98, 21–34. doi: 10.1111/j.1744-7348.1981.tb00419.x

Werker, A. R., Dewar, A. M., and Harrington, R. (1998). Modelling the incidence of virus yellows in sugar beet in the UK in relation to numbers of migrating *Myzus persicae*. *J. Appl. Ecol.* 35, 811–818. doi: 10.1046/j.1365-2664.1998.355340.x

Whalon, M. E., and Smilowitz, Z. (1979a). GPA-Cast, a computer forecasting system for predicting populations and implementing control of the Green peach aphid on potatoes. *Environ. Entomol.* 8, 908–913. doi: 10.1093/ee/8.5.908

Whalon, M. E., and Smilowitz, Z. (1979b). Temperature-dependent model for predicting field populations of Green peach aphid, *Myzus persicae* (Homoptera: Aphididae). *Can. Entomol.* 111, 1025–1032. doi: 10.4039/Ent1111025-9

Worner, S. P., Tatchell, G. M., and Woiwod, I. P. (1995). Predicting spring migration of the damson-hop aphid *Phorodon humuli* (Homoptera: Aphididae) from historical records of host-plant flowering phenology and weather. *J. Appl. Ecol.* 32, 17. doi: 10.2307/2404412

Wu, W., Piyaratne, M., Zhao, H., Li, C., Hu, Z., and Hu, X. (2014). Butterfly catastrophe model for wheat aphid population dynamics: construction, analysis and application. *Ecol. Modell.* 288, 55–61. doi: 10.1016/j.ecolmodel.2014.05.017

Xia, J. Y., Wang, J., Cui, J. J., Leffelaar, P. A., Rabbinge, R., and van der Werf, W. (2018). Development of a stage-structured process-based predator–prey model to analyse biological control of cotton aphid, *Aphis gossypii,* by the sevenspot ladybeetle, *Coccinella septempunctata*, in cotton. *Ecological Complexity* 33, 11–30. doi: 10.1016/j.ecocom.2017.09.003

Zhang, K., Pan, Q., Yu, D., Wang, L., Liu, Z., Li, X., et al. (2019). Systemically modeling the relationship between climate change and wheat aphid abundance. *Sci Total Environ* 674, 392–400. doi: 10.1016/j.scitotenv.2019.04.143

Zhou, X., Carter, N., and Mumford, J. (1989). A simulation model describing the population dynamics and damage potential of the rose grain aphid, *Metopolophium dirhodum* (Walker) (Hemiptera: Aphididae), in the UK. *Bull. Ent. Res.* 79, 373–380. doi: 10.1017/S000748530001837X
